# Secreted *Giardia intestinalis* cysteine proteases disrupt intestinal epithelial cell junctional complexes and degrade chemokines

**DOI:** 10.1080/21505594.2018.1451284

**Published:** 2018-05-04

**Authors:** Jingyi Liu, Showgy Ma'ayeh, Dimitra Peirasmaki, Britta Lundström-Stadelmann, Lars Hellman, Staffan G. Svärd

**Affiliations:** a Department of Cell and Molecular Biology, Uppsala University, Uppsala, Sweden; b Institute of Parasitology, Vetsuisse Faculty, University of Berne, Berne, Switzerland

**Keywords:** parasite, diarrhea, tight junction, chemokine, intestinal barrier, secretion, cathepsin B, Host-pathogen interactions

## Abstract

Giardiasis is a common diarrheal disease caused by the protozoan parasite *Giardia intestinalis*. Cysteine proteases (CPs) are acknowledged as virulence factors in *Giardia* but their specific role in the molecular pathogenesis of disease is not known. Herein, we aimed to characterize the three main secreted CPs (CP14019, CP16160 and CP16779), which were identified by mass spectrometry in the medium during interaction with intestinal epithelial cells (IECs) *in vitro*. First, the CPs were epitope-tagged and localized to the endoplasmic reticulum and cytoplasmic vesicle-like structures. Second, we showed that recombinant CPs, expressed in *Pichia pastoris*, are more active in acidic environment (pH 5.5-6) and we determined the kinetic parameters using fluorogenic substrates. Third, excretory-secretory proteins (ESPs) from *Giardia* trophozoites affect the localization of apical junctional complex (AJC) proteins and recombinant CPs cleave or re-localize the AJC proteins (claudin-1 and -4, occludin, JAM-1, β-catenin and E-cadherin) of IECs. Finally, we showed that the ESPs and recombinant CPs can degrade several chemokines, including CXCL1, CXCL2, CXCL3, IL-8, CCL2, and CCL20, which are up-regulated in IECs during *Giardia*-host cell interactions. This is the first study that characterizes the role of specific CPs secreted from *Giardia* and our results collectively indicate their roles in the disruption of the intestinal epithelial barrier and modulating immune responses during *Giardia* infections.

## Introduction

*Giardia intestinalis (syn. Giardia duodenalis and Giardia lamblia)* is a protozoan parasite that colonizes the upper small intestines of mammals and is a major cause of waterborne diarrhea worldwide [1,2]. There are eight *G. intestinalis* genotypes or assemblages designated from A to H, of which parasites that belong to assemblages A or B infect humans [3]. The cyst, acquired through the oral-fecal route, is the infectious form of the parasite. It breaks open in the duodenum and jejunum, releasing excyzoites that quickly differentiate to trophozoites [1]. The trophozoites adhere to the apical surface of IECs with an adhesive disc [4]. This close contact and subsequent interaction results in a succession of pathophysiological changes, leading to diarrhea, malabsorption and weight loss [5]. These outcomes manifest clearly in immunocompromised or elderly people and in young children of the developing world [6].

The intestinal epithelial barrier (IEB) works selectively to separate the external environment of the intestinal lumen from underlying host tissues and it is formed by tight and adherens junctions (together known as apical junctional complexes, AJCs) [7,8]. AJCs localize intercellularly creating a seal to prevent the paracellular diffusion of microorganisms and antigens across the epithelium [7,8]. They are composed of transmembrane proteins (e.g. claudins, occludin, junctional adhesion molecules (JAMs)), cytosolic plaque proteins (zonula occludens (ZO) family) and cytosolic regulatory proteins (F-actin, α-actinin) [7]. A perijunctional acto-myosin belt-like ring encircles the apical pole of epithelial cells and it is tightly linked to AJCs. The acto-myosin ring regulates the tight junction structure (e.g. claudins and occludins) and paracellular permeability [9]. *Giardia* increases intestinal epithelial permeability in human patients and in experimentally infected mice [10,11]. The increased intestinal epithelial permeability is due to AJC alterations, epithelial cell apoptosis and arginine starvation [8]. Trophozoite attachment and excretory-secretory products (ESPs) released during infection of IECs are believed to be responsible for the structural changes seen in the AJCs [12–16].

*Giardia* ESPs contain several protease activities as determined by substrate impregnated SDS-PAGE or zymogram gels and proteomics and the main activities belong to the cysteine proteases (CPs) [17–19]. Accumulating data suggest that giardial CPs are involved in disease induction and pathogenesis [20]. BALB/c mice administered ESPs orally exhibited signs of mucosal injury and developed specific humoral immune responses, which were less apparent upon ESPs treatment with E-64, a CP-specific inhibitor [21]. An increase in CP secretion has been seen during host-parasite interactions in vitro [18]. It has been shown that CPs can disrupt cellular junctions, compromising the integrity of the IEB [22]. Recent reports have also shown that CP activities from *Giardia* are able to induce cleavage of the microvillus protein villin [23], cleave the chemokine IL-8 and reduce inflammation [24], affect the bacterial normal flora and biofilm formation [25,26] n and inhibit the growth of intestinal bacterial pathogens [27]. Taken together, these studies show an important role for CP activities during host-*Giardia* interactions. However, the roles of CPs in *Giardia*'s pathogenicity have been investigated using cell extracts and/or CP inhibitors but the function of specific CPs from *Giardia* in the disease mechanism(s) requires further investigations.

The CPs are the most prevalent types of proteases in the *Giardia* WB genome; totally 26 genes with 9 cathepsin B-like, 4 cathepsin C-like and 13 cathepsin K/L-like genes [28,29]. The cathepsin B-like proteases are the most highly expressed cathepsins and many are up-regulated during differentiation and *Giardia*-IEC interactions *in vitro* [30–35]. Specific CPs have been suggested to be involved in excystation (CP1 or CP10217, CP2 or CP14019 and CP3 or CP16779) [36], encystation (CP14109) [29] and degradation of endocytosed proteins (CP14019) [37].

The aim of this study was to identify the major secreted *Giardia* CPs during interaction with IECs and to study their roles during *Giardia* infections. Based on earlier reports of giardial CP activities during host-parasite interactions we hypothesized that the proteolytic activity of the CPs disrupts the AJCs and enables the CPs to pass through the intestinal barrier so they can degrade the chemokines produced by IECs.

## Results

### Indentification of secreted cysteine proteases by gelatin zymogram gels and mass spectrometry

**Figure 1.**
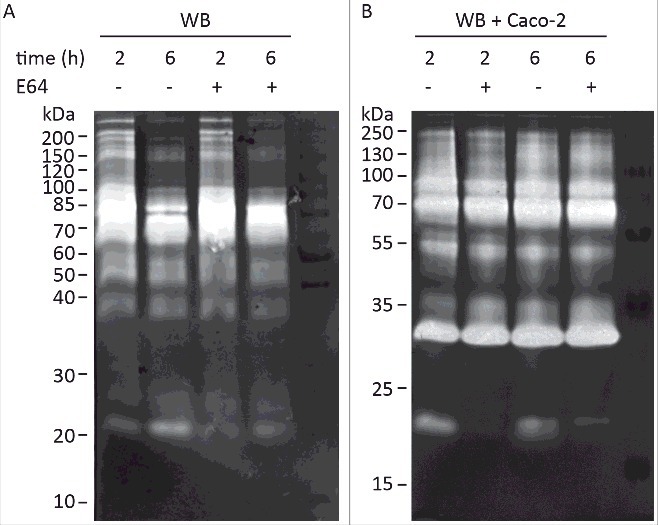
Detection of *G. intestinalis* secreted proteases in the culture supernatant of trophozoites grown alone or in interaction with Caco-2 cell monolayers. Samples of medium were collected after 2 h and 6 h incubation of WB trophozoites at 37°C in DMEM in the absence (A) and presence (B) of Caco-2 monolayer, respectively. The presence of cysteine proteases was detected in concentrated culture supernatants from both axenic and co-cultures by using E64 to inhibit the activities of cysteine protease. For E64 pretreated samples, 100 µM of E64 was used in pre-incubation with ES products at 37°C for 30 min before the electrophoresis. The protein ladders are indicated on the left (kDa).

Several earlier studies have reported CP activities as part of *Giardia* ESPs on zymogram gels but the specific proteases have never been identified [17–19]. We have recently identified several giardial and human CPs in the culture supernatant during Giardia host-cell interactions using LC-MS/MS (Table S1) [38]. Here we decided to complement this by identifying the main secreted CPs on zymogram gels, especially the activities that increase during host-parasite interactions [18]. Secreted proteins of *Giardia* trophozoites (human isolate WB, assemblage A) were collected in a serum-free medium for 2 h and 6 h in the presence or absence of differentiated Caco-2 cells and separated on zymogram gels to detect protease activities. Analyses of the zymogram gels revealed proteolysis bands with molecular weights ranging from 20–200 kDa (Fig. 1), amongst which proteases of 60–100 kDa exhibited the most pronounced proteolytic activity. In the absence of Caco-2 cells, proteolytic activities were more pronounced in samples collected from 2 h of incubation and some bands around 20 kDa are slightly inhibited by treatment with the cysteine protease inhibitor E64 (Fig. 1A). In the presence of Caco-2 cells (Fig. 1B), proteolytic patterns were similar to those in axenic culture samples, but the intensities of all bands were stronger, suggesting an increase in the proteolytic activity upon incubation with host cells. We also noticed the appearance of new bands around 30–35 kDa in molecular weight during the interaction (Fig. 1B). The activities are not inhibited by E64 and it is most likely one or several of the released human proteases (Table S1) since it can be seen when only Caco-2 cells are analyzed. The proteolytic activity of the bands around 20 kDa was significantly inhibited when ESPs from co-culture with either *Giardia* isolate were treated with E64 (Fig. 1B). These bands were excised from the non-E64 treated lane (Fig. 1B) of the WB trophozoite-host cell interaction and analyzed using mass spectrometry (Materials and Methods). This identified a few tryptic peptides that belong to three giardial clan CA cathepsin B-like CPs: CP1 to CP3 or CP10217, CP14019, and CP16779 (Table S2). It should be noted that one peptide (N-NSWGPDWGEDGYFR-C) can be found in both CP14019 and CP16779, but also in another CP, CP16160. Thus, it is possible that CP16160 is also secreted. This has been verified in our recent proteomics study of giardial ESPs where CP14019, CP16160 and CP16779, were found to be the main CPs released during the interaction between trophozoites and IECs (Table S1) [38].

### Expression and localization of the secreted CPs

**Figure 2.**
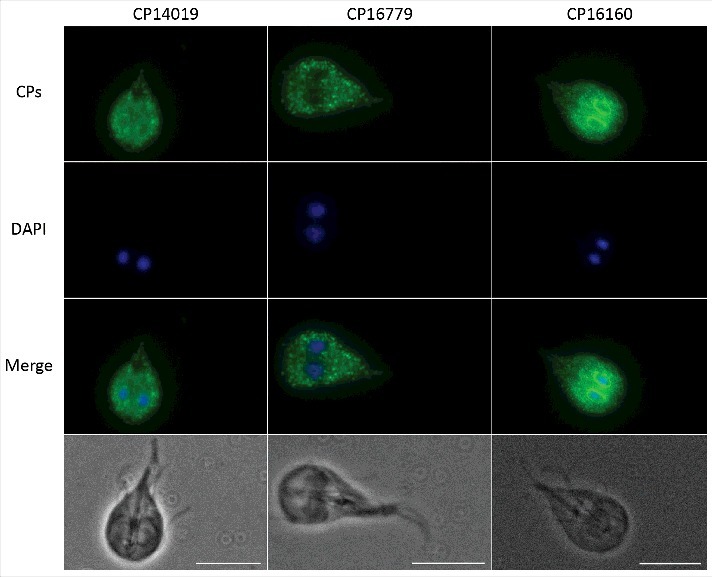
Localization of *G. intestinalis* cathepsin B-like cysteine proteases. The localization of 3xHA epitope-tagged cathepsin B-like cysteine proteases (CP14109, CP16160 and CP16779) by immunofluorescence microscopy. Transfectants were detected with a mouse anti-HA antibody conjugated to Alexa Fluor 488. Images were taken by a Zeiss Axioplan II Imaging fluorescence microscope and analyzed by ZEN 2.1 software. Bar = 10 µm.

There are 8 genes in the WB genome that encode clan CA cathepsin B-like CPs with a mature size of 20–25 kDa [29]. The genes encoding the four identified secreted CPs localize to chromosomes 1 (10217), 3 (CP16160) and 4 (CP14109 and 16779) in the *G. intestinalis* WB isolate (www.giardiadb.org) and they can be found in syntenic regions in parasites from assemblages B (GS isolate) and E (P15 isolate). All proteins have predicted signal- and propeptides but the N terminal of CP16160 in the WB genome is most likely wrongly annotated and should start with MIGASLLLGAV (www.giardiadb.org). CP14019 shows 80% identity to CP16779 and 50% identity to CP10217 and CP16160. The structures of the pro- and mature proteases were predicted using iTasser and Phyre2 and the structural models aligned well with known cathepsin B structures (Fig. S1 to S3, Table S3 to S5). CP10217, CP14019 and CP16779 are most similar to *Trypanosoma congolese* procathepsin (PDB: 5FPW), whereas CP16160 is more similar to the human procathepsin B (PDB: 7PCK). A conserved cysteine (position 26 in CP16160) and histidine (position 165 in CP16160), predicted to be 3.7Å apart in a hydrophobic pocket on one side of the protein, most likely make up the catalytic dyad of the enzymes (Fig. S4). Expression data shows that the four CPs are among the most highly expressed genes in *Giardia* trophozoites and expression increases in encystation [39]. Interestingly, CP14109 and CP16779 are up-regulated at the RNA level upon host cell contact [30–32] and CP16160 is more highly expressed at the protein level in virulent *Giardia* strains [34]. Earlier studies have localized CP14019 and CP10217 to the ER and cytoplasmic vesicles, using GFP fusions and polyclonal antibodies [37,39]. We produced transfectant cell lines expressing C-terminally epitope-tagged constructs of the three cysteine proteases CP16779, CP14019 and CP16160 in *G. intestinalis* and imaged them by immunofluorescence microscopy. The results showed that all three proteins localized to an ER-like and punctuate vesicle-like structures within the cytoplasm (Fig. 2), similar to what has been seen earlier with CP14019 and CP10217 [37,39]. Thus, all four CPs localize to the same cellular structures in *Giardia* trophozoites.

### Characterization of recombinant CP14019, CP16160 and CP16779

**Figure 3.**
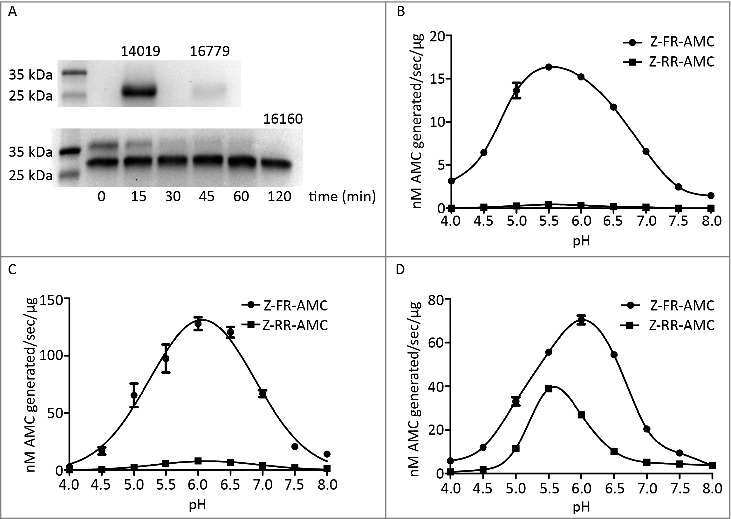
Recombinant *G. intestinalis* secreted cysteine proteases were expressed, purified and characterized. (A) SDS-page gel showed that both 14019 and 16779 were expressed as the mature form of 28 kDa whereas 16160 was expressed as a combination of a zymogen of 35 kDa and a mature form of 28 kDa. 16160 auto-activated to the mature form after 120 min incubation in sodium acetate buffer (pH 5.5). (B-D) pH profiles of CP14019 (B), CP16779 (C) and CP16160 (D) against the fluorogenic substrates Z-FR-AMC and Z-RR-AMC showed that the pH optimum was 5.5-6.0 for activity against both substrates.

We decided to characterize CP14019, CP16160 and CP16779 further since they have interesting expression patterns during differentiation, host cell interactions and in virulent strains (see above). The three CPs were expressed in *P. pastoris* and purified using Ni-columns that bind the polyhistidine-tag in the recombinant proteins (Fig. 3A). The recombinant CP14019 and CP16779 were found to be auto-activated to the mature form (28 kDa) during the purification process (Fig. 3A), whereas the expressed CP16160 was found in two forms, 28 kDa and 37 kDa, during purification but the proenzyme auto-cleaved to the mature form (28 kDa) after 2 hours incubation in a sodium acetate buffer (pH 5.5) (Fig. 3A).

To measure the activity of mature enzymes, the activity profile of each recombinant enzyme was examined against the artificial substrates Z-FR-AMC and Z-RR-AMC that were used earlier with CP14019 [29]. All CPs were found to be more active under acidic condition. Specifically, the optimal pH for CP14019 and CP16779 was found to be 5.5 and 6, respectively, for both substrates (Fig. 3B and C, Fig. S5) whereas CP16160 cleaved Z-FR-AMC at an optimal pH of 6.0 and Z-RR-AMC at a pH of 5.5 (Fig. 3D, Fig. S5). We then measured the kinetic parameters of the enzymes against Z-FR-AMC and Z-RR-AMC under the determined optimal pH conditions (Fig. S6). The same amount of enzyme was incubated with various concentrations of substrates, fluorescence units were recorded and the kinetic parameters of the enzymes were calculated. The K_m_ and K_cat_/K_m_ values for the two fluorescent substrates are presented in Table 1. The results showed that CP16779 exhibited the lowest K_m_ values and highest K_cat_/K_m_ for the Z-FR-AMC substrate. For the Z-RR-AMC substrate, the K_m_ and K_cat_/K_m_ values were very similar for the three enzymes (Table 1), with the K_m_ value for CP14019 being the lowest. The three CPs have a preference for Z-FR-AMC over Z-RR-AMC but the cleavage rates are more similar for CP16160 (Fig. 3 and Fig. S6). These results show that while the three enzymes can use similar substrates, the reaction dynamics might vary between them, including the amount of substrate required to reach the same reaction speed and substrate preference.

**Table 1.** Kinetics parameters for secreted cysteine proteases. Recombinant enzymes were incubated with various concentrations of fluorogenic substrates under the optimal pH conditions and fluorescence units were recorded at 37°C over time. Data was calculated using Michaelis-Menten kinetics in the Graphpad Prism 5.0 software.

	Z-FR-AMC			Z-RR-AMC		
Enzyme	Optimal pH	K_m_ (µM)	K_cat_/K_m_ (M^−1^*S^−1^)	Optimal pH	K_m_ (µM)	K_cat_/K_m_ (M^−1^*S^−1^)
CP14019	5.5	3.17± 0.34	(1.79∼2.37)X 10^5^	5.5	5.11± 1.11	(5.41∼9.84)X 10^3^
CP16779	6	0.96± 0.24	(1.02∼2.06)X 10^6^	6	5.40± 0.75	(1.05∼1.48)X 10^4^
CP16160	6	9.40± 1.46	(4.30∼6.77)X 10^4^	5.5	5.60± 1.00	(5.86∼9.61)X 10^4^

### The epithelial barrier breakdown by *G. intestinalis* ESPs and recombinant cysteine proteases

**Figure 4.**
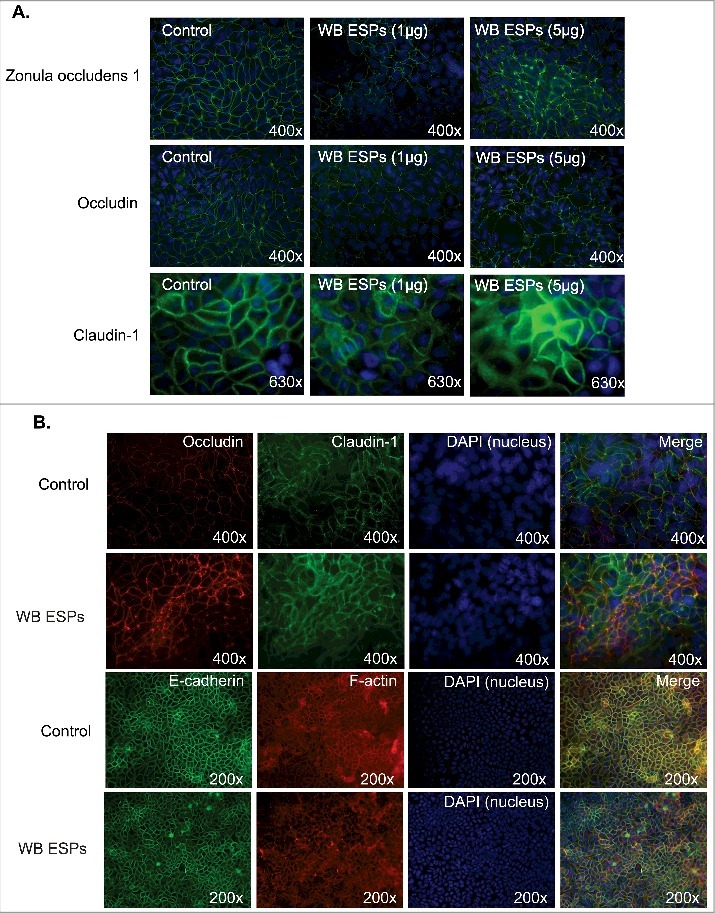
Effect of ESPs on apical junction complexes. A. Intestinal epithelial cells (Caco-2) were treated with 1 and 5 ug of concentrated ESPs from *Giardia* WB trophozoites in axenic culture in serum-free medium. The localizations of Zonula Occludens-1 (ZO-1), Occludin and Claudin-1 were studied using specific antibodies. B. Intestinal epithelial cells (Caco-2) treated with non-concentrated ESPs from *Giardia* trophozoites in axenic cultures in serum-free medium. The localizations of Occludin, Claudin-1, E-cadherin and F-actin were studied using specific antibodies. Images were taken by a Zeiss Axioplan II Imaging fluorescence microscope and analyzed by ZEN 2.1 software.

*Giardia* trophozoites affect the integrity of the AJCs between IECs and the tight junction proteins occludin and claudin-1 and -4 have been shown to be delocalized and/or degraded during host parasite interactions [15,40]. It has also recently been shown that *Giardia* ESPs bind to the intercellular junctions between IECs [38]. To assess whether the interaction or binding of ESPs to intercellular junctions affected their integrity, IECs were exposed to ESPs (Fig. 4). We collected ESPs from axenic cultures of WB trophozoites in a special serum-free medium, which is optimized for trophozoite viability (Materials and Methods). In the first experiments (Fig. 4A) the ESPs were concentrated and 1 and 5 μg /ml ESPs was added to the IECs in chamber slides. Immunofluorescence was used to identify structural changes in the IEC intercellular junctions. The results showed flocculation and reorganization of ZO-1, occludin and claudin-1 in IECs exposed to 1 and 5 μg (per ml) of *Giardia* ESPs (Fig. 4A). Similar results were obtained for occludin and claudin-1 after addition of non-concentrated ESPs (Fig. 4B). We could also detect changes in F-actin localization, whereas E-cadherin did not change (Fig. 4B). This shows that the *Giardia* ESPs affect AJCs between IECs.

**Figure 5.**
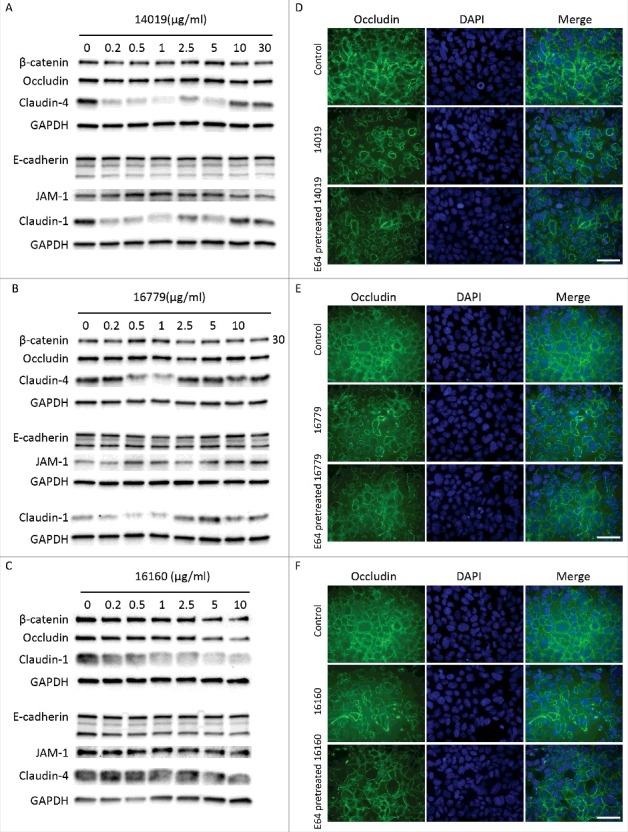
*G. intestinalis* secreted cysteine proteases have effects on epithelial barrier proteins. A, B, C. Levels of tight junction and adherence junction proteins in Caco-2 whole cell extracts were examined after 24 h of recombinant cysteine proteases treatment (A, 14019; B, 16779; C, 16160), respectively, by Western blot analysis, with GAPDH used as a loading control. Barrier proteins were detected with a mouse or rabbit anti-barrier protein antibody and then incubated with a HR- conjugated mouse or rabbit antibody. D E, F. The localization of occludin in Caco-2 cells visualized by immunofluorescence microscopy. Caco-2 monolayers were incubated with 2.5 µg/ml of 14019 (D), 16779 (E), 16160 (F) in the absence or presence of E64 (10 µM) for 24 h, followed by fixation of PFA. Occludin was detected with a mouse anti-occludin antibody and then incubated with an Alexa Fluor 488-conjugated mouse antibody. Images were taken by a Zeiss Axioplan II Imaging fluorescence microscope and analyzed by ZEN 2.1 software. Bar = 50 µm.

To assess the effects of the three CPs on the AJCs, we used Western blotting and immunofluorescence to examine the degradation and distribution of junctional proteins after treating Caco-2 cells with recombinant CPs. Recombinant CP14019 and CP16779 were able to degrade claudin-1 and -4 after 24 h of incubation with Caco-2 cells, with the degradation being more pronounced at lower enzyme concentrations (< 1 μg/ml) (Fig. 5A and B, Fig. S7). Both CPs could also cause a slight degradation of β-catenin, occludin and JAM-1, but the degradation pattern was inconsistent with the amount of CPs added. No clear changes in the levels of E-cadherin were seen with both CPs. In the case of CP16160, the degradation of the aforementioned junctional proteins occurred in a dose-dependent manner (Fig. 5C, Fig. S7). Since we did not reach a conclusive result on the ability of the CPs to degrade E-cadherin, we tested their ability to directly degrade human recombinant E-cadherin (rhE-cadherin) using different concentrations of the proteases and incubation periods (Fig. S8). Here, we could clearly see the degradation of rhE-cadherin at enzyme concentrations as low as 0.05 μM and within 5 minutes incubation with this protein. We could also show that recombinant occludin is digested by the CPs (Fig. S9). Overall, the results show that recombinant CPs are able to degrade junctional complex proteins required for the integrity of the intestinal epithelial barrier. The reduced digestion of certain AJC proteins can be due to limited access of the recombinant CPs to the proteins. Furthermore, immunofluorescence images showed reduced levels and re-localization of the tight junction proteins occludin and claudin-4, as well as the adherence junction proteins, E-cadherin and beta-catenin, after 24 h of incubation with the recombinant CPs (Fig. 5D–F, Fig. S10–S13). A disruption in the integrity of the Caco-2 cell monolayer was also observed and these effects were reduced with CPs inhibited by E-64 (Fig. S10–S13). These findings indicate that the CPs released by *Giardia* might play a role in compromising the integrity of the intestinal epithelial barrier.

### Chemokine degradation by ESPs and recombinant CPs

**Figure 6.**
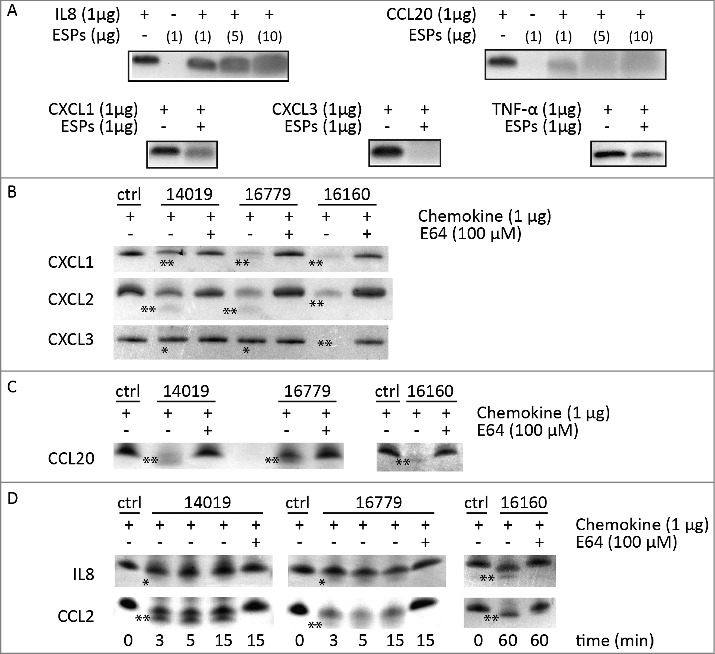
*G. intestinalis* ESPs and cysteine proteases have a proteolytic effect on chemokines. A. Digestion of chemokines with ESPs collected from axenic *Giardia* WB cultures. B-D. Cleavage of chemokines by recombinant cysteine proteases was performed as described in Methods. 100 µM of E-64 was routinely added to the proteases for 30 min at 37°C. Samples were analyzed by 16.5% Tris-Tricine gel under reducing condition. **represents “clear degradation of chemokines”, *represents “uncertain cleavage of chemokines”.

Recent data have shown that CP activities from *Giardia* can degrade chemokines like IL-8 and reduce inflammation [24]. To follow up this observation, we tested if *Giardia* ESPs are capable of degrading cytokines/chemokines that are induced during *Giardia*-IEC interactions [41]. We incubated concentrated *Giardia* ESPs with different pure cytokines and chemokines and electrophoresed the reaction mixture on SDS-PAGE gels to visualise potential degradation (Fig. 6A). Incubation of IL-8 with 1 μg of ESPs from each isolate resulted in the appearance of fuzzy bands on stained gels (compared to control), indicating their cleavage/partial degradation (Fig. 6A). IL-8 degradation manifested clearly (i.e. fainter and fuzzier bands) with increasing amounts of ESPs (5 μg and 10 μg, Fig. 6A). For CCL20, a fuzzy band could already be seen upon incubation with ESPs of WB isolate (1μg) and it increased with higher amounts of ESPs (Fig. 6A). CXCL1 and TNF-α appeared as fuzzy bands upon incubation with 1μg of ESPs from each isolate (i.e. partial degradation) (Fig. 6A). ESPs of the WB isolate completely degraded CXCL3 (Fig. 6A). These results demonstrate that ESPs of *Giardia* are capable of degrading or cleaving different cytokines/chemokines. Thus, *Giardia* ESPs might exert immunomodulatory functions represented by the attenuation of inflammatory responses induced by the chemokines produced by IECs.

To test whether the chemokines expressed by Caco-2 cells during *Giardia* infection [41] can be potential substrates for CPs, we incubated 1 µg of chemokines with active mature enzymes in the presence or absence of E-64 and examined their cleavage/degradation by gel electrophoresis (Fig. 6, Table S6). Both CP14019 and CP16779 cleaved the chemokines, CXCL2, CCL20 and CCL2 as evidenced by the appearance of two lower molecular weight bands on the gel after protease treatment compared to controls (i.e. untreated chemokines) (Fig. 6B–D). In case of CXCL1, fuzzier bands appeared on the gel indicating partial degradation whereas for IL-8, a fuzzy lower molecular band appeared under the chemokine band indicating cleavage. No CXCL3 degradation could be seen with CP14019 and CP16779, indicating substrate specificity in the mode of action of the two CPs. In case of CP16160, all tested chemokines were either degraded (e.g. CXCL1-3) or cleaved (e.g. CCL2, CCL20 and IL-8) (Fig. 6B–D). For all the CPs tested, E-64 treatment abolished their ability to degrade the chemokines (Fig. 6B–D). While these results show that *Giardia* CPs exhibit selectivity and differential ability to degrade inflammatory chemokines, they also highlight the potential of *Giardia* released CPs to modulate inflammatory responses.

## Discussion

**Figure 7.**
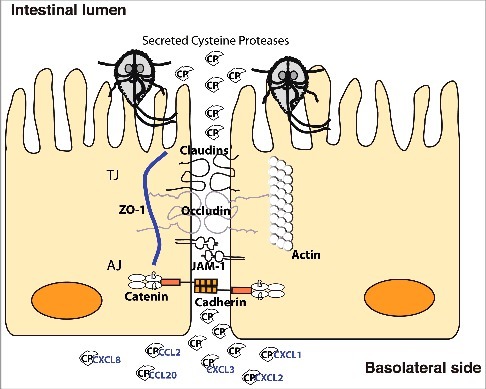
Model of cathepsin B cysteine proteases function during *Giardia*-host cells interactions. *Giardia* cathepsin B cysteine proteases released during host-parasite interaction have proteolytic activity and are capable of destroying the junctional complexes (TJs and AJs). The CPs can pass the epithelial barrier and degrade chemokines that are induced by intestinal epithelial cells and released on the basolateral side in response to *Giardia* infection.

Cathepsin B-like protease activities from *Giardia* have recently been shown to be important in host-pathogen interactions in several different studies [23–27]. However, the specific proteases have not been identified. Herein, we focused on three CPs that we identified in the medium of *Giardia*-host cell interactions, characterized them and identified their potential involvement in disease mechanisms. Our data suggest that the CPs are involved in destruction of the intestinal epithelial barrier and in chemokine degradation (Fig. 7).

First, we examined the proteolytic activity in the medium of co-culture and compared the banding pattern to that of axenic culture. We showed proteolytic bands on zymogram gels with molecular weights ranging between 20 and 200 kDa, with an increase in the intensity of CP bands specifically on contact with IECs. Similar results were previously reported by Rodríguez-Fuentes et al. [18] who demonstrated that the released CPs play a role in trophozoite attachment to IECs. Previous reports have shown the up-regulation of CP14019, CP16779 and CP16160 at the RNA and protein levels during host-parasite interaction *in vitro* and recently they were shown to be released into the co-culture medium [30–32,34,35,38,42]. Here we identified the same CPs in the zymogram gel bands. Secretion is further supported by the presence of secretion signal peptides in these CPs and localization to vesicle-like structures dispersed through the cytoplasm and ER, which have been shown to be involved in protein uptake and release [43].

To characterize the identified CPs, we used *P. pastoris* as an expression system through which the proper conditions required for enzyme activity (e.g. correct folding and glycosylation) are attained. The CP14019 and CP16779 auto-activated during the purification process whereas the CP16160 was present in both the zymogen and active forms, which have been previously seen during the proteolytic maturation of pro-cathepsins [44]. Activation occurs spontaneously or upon exposure to acidic environment and it is mediated by the pro-peptide functioning as a chaperon to assist protein folding and controlling the protease activity [43,44]. The recombinant CPs displayed an optimal activity at an acidic pH (e.g. 5.5-6), the conventional pH for this class of enzymes [29,37,45] and a preference for the fluorescent substrate FR-AMC. These attributes have been previously ascribed to the conformational change in the binding site (i.e. S2 pocket, Fig. S4) upon enzyme maturation, the change in the hydrogen ion concentration (i.e. pH) and/or the activity of the mature enzyme being regulated by the pro-peptide [29,39]. How these CPs behave when released all together from *Giardia* upon contact with host cell and how their interaction with each other and host cells affect their activity is an interesting subject for further research.

The maintenance of the intestinal epithelial barrier integrity is of paramount importance to prevent to the follow of microbes and antigens into the underlying tissue [7,8]. This is achieved by forming a seal-like structure through the specific organization of AJC proteins. Their arrangement, however, is reorganized during microbial infections compromising the integrity of epithelial barrier and increasing its permeability. This is the case in *Giardia* infections where the re-arrangement of ZO-1, α-actinin, occludin and F-actin has been reported in *in vitro* studies together with the increase in the permeability of epithelial cell monolayer [13–16,22]. The expression level of claudin-1, the transmembrane protein responsible for the sealing properties of tight junctions, decreased in chronic giardiasis patients [11]. Therefore, it has become evident that *Giardia* infections affect the integrity of intestinal epithelia by reorganizing proteins of the AJCs. In this report, we provided a further insight into the ability of *Giardia* ESPs, namely CPs, to replicate these effects and expanded the range of apical junctional complexes proteins affected by *Giardia* CPs to include claudin-4, β-catenin and E-cadherin (Fig. 7). We also showed that these effects are brought about by either the degradation or re-distribution of the proteins above, which overall disrupts the integrity of epithelial sheath. Certain AJC proteins were less affected after addition of recombinant proteins to the IECs compared to the degradation of recombinant proteins (E-cadherin) or after addition of parasites. The parasites attach strongly with the adhesive disc [4] and it is possible that this interaction contribute to the destruction of the AJCs. In conjunction with results from previous reports [19,46,47] showing that parasite CPs are capable of degrading a wide range of substrates including collagen I, an important component of the extracellular cellular matrix, these findings collectively indicate that *Giardia* CPs play an important role in disease induction.

The genes encoding different chemokines are induced in IECs upon *Giardia* infection *in vitro* [41]. The chemokines induced include CCL2, CCL20 and CXCL1-3, amongst which CCL20 exhibited the highest fold change in RNA levels during the first 90 minutes of interaction [41]. This unique chemokine profile targets different populations of immune cells like dendritic cells, T cells, and B cells by CCL20, neutrophils by CXCL1 to CXCL3, and macrophages and T-cells by CCL2 to the site of infection [41]. However, the amounts of CCL20 produced and released into the medium of interaction were low (< 50 ng/ml) compared to the transcriptional level, indicating post-transcriptional regulation of CCL20 levels. In fact, a similar mechanism has been reported for IL-8 in another study, where the degradation of IL-8 by *Giardia* CPs resulted in the attenuation of neutrophil chemotaxis in assays using a cell culture model of infection and *ex vivo* human biopsies [24]. Herein, we corroborate the findings above by showing that the recombinant CPs were able to cleave/degrade CCL20 and IL-8, an effect that was abolished by inhibiting CP function with E-64 treatment. Not only this, but CCL2 and CXCL1-3 could also be cleaved/degraded by the expressed CPs, although differences in substrate specificities were observed (Fig. 7). The substrate specificity is not uncommon and could be attributed to structural differences between the recombinant CPs, as observed in our structural analysis (File S1), hence their ability to interact with different substrates. In *Entamoeba histolytica*, CPs are important for pathogenesis and a recombinant CP, named EhCP2, was able to cleave the chemokines CCL-2, CCL-13 and IL-8, impairing their function in chemotaxis assays with monocytes and granulocytes [45,48]. Despite this, it is worth noting that EhCP2 had an opposite effect on a less active variant form of IL-8 that has 5 extra amino acid residues in the N-terminal sequence [48]. This could also imply that some CPs contribute in the maturation of certain cytokines and thus favoring the induction of pro-inflammatory microenvironment for nutrient acquisition and dissemination. It will be interesting to identify the cleavage motifs for the CPs above and test whether the cleaved chemokines become ineffective in chemotaxis to fully assess the impact of *Giardia* CPs on modulation of immune response. Combining the data from studies of CP degradation of junctional proteins with the data from chemokine degradation makes it possible to propose a model (Fig. 7) for how the combined activities can interact to reduce the effect of chemokines that are induced in IECs during host parasite interactions. The model can partly explain the low levels of inflammation seen during giardiasis [49–51] and it can also explain the observed problems with the epithelial barrier during giardiasis [8]. Thus, at the same times as the parasite reduces inflammation it can also create diarrhea and post-infectious problems like inflammatory bowel syndrome (IBS) and food allergies. This suggests that the identified CPs are key virulence factors in *Giardia*.

In summary, we have identified and expressed the three main CPs released by *Giardia* during interaction with host intestinal epithelial cells *in vitro*. The recombinant CPs displayed characteristics of cathepsin B in terms of their activity and varied in their kinetics and substrate preference. The expressed CPs were able to degrade and reorganize junctional proteins and degrade a range of chemokines, indicating potential roles in the disruption of intestinal epithelial barrier and immune modulation. Further studies will focus on characterizing these proteases in *Giardia* during infections in animal models and humans and it can hopefully identify their specific role in disease.

## Materials and methods

### Parasite cultivation and cell culture

*G. intestinalis* isolates, WB clone C6 (ATCC number 50803, assemblage A) and GS (ATCC number 50581, assemblage B), were used in this study. Trophozoites of each isolate were propagated in modified TYI-S-33 medium [52] supplemented with 10% heat inactivated bovine serum (Gibco, cat no. 26170043) at 37°C until reaching 80% confluence, then used in the experiments.

The human intestinal epithelial colorectal adenocarcinoma cell line, Caco-2 (ATCC HTB-37, passage no. 10–16), was cultured and maintained in Dulbecco´s modified Eagle´s medium (DMEM) (Sigma-Aldrich, cat no. D6546) supplemented with 10% heat-inactivated fetal bovine serum (HI-FBS) (Thermo Fisher Scientific, cat no. 10100147), 100 µg/ml streptomycin and 100 U/ml penicillin (Thermo Fisher Scientific, cat no. P0781), 200 mM L-glutamine (Thermo Fisher Scientific, cat no. 35050061) and 100 µM non-essential amino acids (Sigma-Aldrich, cat no. M7145). Cells were seeded into T25 tissue culture flasks (Sarstedt, cat no. 83.3910, 12-well plates (Corning, cat no. 3513) and Nunc Lab-Tek II Chamber Slide (Sigma-Aldrich, cat no. C7057) and incubated in complete DMEM. Cells in T25 flasks were left to differentiate for 14–21 days post-confluence and the 12-well plates and chamber slides were maintained for 3 days post confluence before being used in the experiments. For all cell cultures, the media were changed twice weekly and incubated at 37°C in a 10% CO_2_ humidified tissue culture incubator.

### Collection *Giardia* excretory-secretory products (ESPs)

To collect *Giardia* ESPs, TYI-S-33 media in axenic cultures (48 h) were decanted and the tubes were washed three times with warm PBS to remove non-attached or dead trophozoites. Thereafter, tubes with adherent trophozoites were replenished with serum-free M199 medium (Thermo Fisher Scientific, cat no. 11043023) supplemented with 6 mM ascorbic acid (Sigma-Aldrich, cat no. A4544) and 6 mM L-cysteine (Sigma-Aldrich, cat no. C7880) (pH 7.2, at 37°C) and incubated for 2 h and 6 h. To collect ESPs in co-culture with Caco-2 cells, parasite cultures were treated as above but with the inclusion of cold shock step (i.e. wet ice) to detach trophozoites for cell counting and resuspension in the media above. Trophozoites were added to pre-washed Caco-2 culture flasks (3 x, warm PBS) in a 3:1 (parasite: IECs) ratio and incubated in a tissue culture incubator for 2 h and 6 h (37°C, 10% CO_2_). Incubation of trophozoites and IECs in the modified M199 medium did not affect their viability as reported in [32]. In the end of all incubations, culture media were collected and centrifuged at 500 × g for 10 min at 4°C to pellet trophozoites and the supernatants were filtered sterilized (0.22 μm) and concentrated using Amicon Ultra-15 Centrifugal Filter Units (Merck Millipore, cat no. UFC900308) with a 3 kDa cut-off. The concentrates were aliquoted and stored at −80°C until use. It should be noted that this isolation method of ESPs also enrich for released vesicles like exosomes and microvesicles [38].

### Detection of protease activity using gelatin zymograms

Before running samples on a zymogram gel, protein concentration was estimated using Qubit protein assay (Thermo Fisher Scientific, cat no. Q33211) according to the manufacturer's protocol. Briefly, ESP samples (30 µg each) were incubated with an equal volume of non-reducing electrophoresis buffer for 15 min at room temperature then loaded into a 10% SDS-PAGE gels copolymerized with 0.1% gelatin and electrophoresed at 100 V, 4°C (non-reducing conditions) until the dye reached the bottom of the gel. Gels were washed three times for 15 min in 2.5% Triton X-100 solution (Sigma-Aldrich, cat no. X100), followed by incubation in a zymogram developing buffer (Thermo Fisher Scientific, cat no. LC2671) overnight at 37°C. Next day, gels were stained with Coomassie Blue and destained in water. Proteases appeared as white bands against a blue background. To identify CPs, samples were treated with 100 µM of trans-Epoxysuccinyl-L-leucylamido (4-guanidino)butane (E64) (Sigma-Aldrich, cat no. E3132-1MG) for 30 min at 37°C before running on the zymogram gel. CPs were identified by the inhibition of protease band activities when compared to untreated gels. Bands identified with CPs were excised from the gels and sent for mass spectrophotometry analysis to identify corresponding peptides.

### Cysteine protease bands and mass spectrometry analysis

Proteomics was performed essentially as described earlier [38]. Proteins were reduced, alkylated and in-gel digested by trypsin, dried and resolved in 15 μL 0.1% FA (formic acid). Peptides were separated in reversed-phase on a C18-column and electrosprayed on-line to an LTQ Orbitrap Velos Pro ETD mass spectrometer (Thermo Finnigan). Tandem mass spectrometry was performed applying CID and it was performed at proteomics core facility at SciLifelab, Uppsala, Sweden.

Database searches were performed using the Sequest algorithm, embedded in Proteome Discoverer 1.4 (Thermo Fisher Scientific), towards a FASTA database containing proteins from *Giardia*, downloaded from giardiadb.org/common/downloads/release5.0/GintestinalisAssemblageA/. The search criteria for protein identification were set to at least one matching peptide of 95% confidence level per protein. In addition, database searches against human proteins in Swissprot were performed in order to identify possible contaminations.

### Cloning of cysteine proteases into plasmids and transfection

Genes were amplified with their endogenous promoter regions using the forward primer 14019-5′: CCCACGCGTCAAACTCTTCACAGAGGCCGCCTGGATCC and the reverse primer 14019-3′: GGGCGGCCGCTCATCGAAGAAGCCCGCATAGGCCTGC, the forward primer 16160–5′: GGTCTAGACAGGCAAGTGAAGAAACCGC and the reverse primer 16160–3′: CCGCGGCCGCTCTACGTCTGCATAGTGGAAGCC, the forward primer 16779-5′: CCCACGCGTGACACCAAGTTGTGTCTTCCTGTAGG and the reverse primer 16779-3′: GGGCGGCCGCTTCTCAAAGAACCCACCTATTACTTGC, and cloned into the episomal pPAC-3xHA-C vector53 using *Xba* I/*Not* I restriction sites for CP16160 and MluI/NotI restriction sites for CP14019 and CP16779. Electroporation was performed as described in [53]. Transgenic parasites were selected by the addition of puromycin (50 μg/ml) approximately 16 h after transfection and stable transfectants were generally obtained after a week.

### Localization of cysteine proteases

Transfected cells were prepared for immunofluorescence microscopy as described [54] with some adjustment. Briefly, trophozoites were harvested by chilling on ice for 10 min, washed twice in ice-cold PBS, and fixed with 2% paraformaldehyde (PFA) (Thermo Fisher Scientific, cat no. 28906) at 37°C for 20 min in a pre-warmed moisture chamber, followed by 10 min washing in 100 mM glycine prepared in PBS. Fixed cells were permeabilized with 0.2% Triton X-100 (Sigma-Aldrich, cat no. X100) in PBS for 30 min and blocked at room temperature for 1 h in 2% bovine serum albumin (BSA) (VWR, cat no. 441555J) prepared in PBS. The anti-HA mouse monoclonal antibody conjugated to Alexa fluor 488 (BioLegend) was diluted 1:250 in PBS containing 2% BSA and 0.05% Triton X-100 (Sigma-Aldrich), incubated with fixed cells for 1 h at room temperature. Images were taken using a Zeiss Axioplan II Imaging fluorescence microscope (Carl Zeiss Light Microscopy, ZEISS) and analyzed by ZEN 2.1 software.

### Transformation and expression of secreted CPs in *Pichia pastoris*

The CP14019, CP16160 and CP16779 genes were amplified by PCR from genomic DNA extracted from *Giardia* WB isolate using the forward primer 14019-5′: GGGAATTCGAGCTTAACCACATCAAGTCCC and the reverse primer 14019-3′: CCTCTAGATCAATGATGATGATGATGATGCTCATCGAAGAAGCCCGCATAGG, the forward primer 16160–5′: GGGAATTCTCTTTGTCCGCGCTACCTCTCA and the reverse primer 16160–3′: CCTCTAGATCAATGATGATGATGATGATGCTCTACGTCTGCATAGTGGAAGCC, the forward primer 16779-5′: GGGAATTCGAGCTTAACCACATCAAGTCCCTGAATCCC and the reverse primer 16779-3′: CCTCTAGATCAATGATGATGATGATGATGGTTCTCAAAGAACCCACCTATTACTTG. The amplicons were cloned into the *EcoR* I/*Xba* I sites of the *Pichia pastoris* expression vector pPICZαA in frame with a poly-histidine tag added to the C-terminal. The plasmids were linearized by *Sac* I or *BstX* I and introduced into *P. pastoris* by electroporation (GenePulser XCell, Bio-Rad) according to the provider's protocol. Transfectants were screened by growth on YPD + 200 µg/ml of Zeocin (Thermo Fisher Scientific, cat no. R25005) and confirmed by Sanger sequencing.

### Purification of CP14019, CP16160 and CP16779

Proteins expression was induced in *P. pastoris* growth during under 3 days period during which 100% methanol was added every 24 h to maintain protein expression (0.5% methanol final concentration). After 72 hours, culture supernatants were collected and filtered using 0.2 µm filtration units. Recombinant proteins were purified using Nickle-resin columns (Thermo Fisher Scientific, cat no. 88222) followed by desalting. Protein concentrations were determined by Bradford Assay (Bio-Rad, cat. no. 5000205) and purified proteins were aliquoted and stored at −80°C until use.

### Detection of cathepsin cysteine protease activity

The activity of recombinant proteins was determined by measuring their ability to cleave the fluorogenic substrates *N*-carbobenzoxy-phenylalanyl-arginyl-7-amido-4-methylcoumarin (Z-FR-AMC) and *N*-carbobenzoxy-arginyl-arginyl-7-amido-4-methylcoumarin (Z-RR-AMC) (Bachem, cat no. I-1160 and I-1135). The substrates were incubated with recombinant proteins in sodium acetate buffers (pH 4.0-5.5) or basic/dibasic sodium phosphate buffers (pH 6.0-8.0) containing 4 mM DTT and 10 mM EDTA. Protein activity was measured by monitoring the increase in relative fluorescence units (RFU) over time at 37°C using an excitation wavelength of 360 nm and an emission wavelength of 470 nm. For CP16160, recombinant protein was pre-activated in a sodium acetate buffer for 120 min (pH 5.5 at 37°C) prior to starting the assay.

To determine the K_m_ and K_cat_/K_m_ of the enzymes, the fluorogenic substrates Z-FR-AMC and Z-RR-AMC were incubated with the enzymes separately at a range of concentrations under the optimum pH condition and the velocity of these reactions was used to calculate the K_m_ and K_cat_/K_m_. Data were analyzed using the Michaelis-Menten model (Prism 5 software, Graphpad).

### Effects of ESPs and recombinant CPs on AJC proteins

Chamber slides with differentiated Caco-2 cells were used to assess the effect of ESPs on AJC proteins. Briefly, IECs grown in Nunc Lab-Tek II chamber slides were washed (2x, PBS) and incubated with fresh complete DMEM (1 h), followed by the addition of media containing ESPs from axenic cultures and further incubation for 6 h (37°C, 10% CO_2_). Concentrated ESPs from axenic cultures were quantified using the Qubit protein reagent and added to IECs at 1, 5 or 10 μg/ml. At 6 h, IECs were washed with PBS and fixed with 4% paraformaldehyde prepared in PBS (Pierce, Thermo Fisher Scientific) for 15 min at RT. Fixative was washed off (3x, PBS) and IECs were blocked for 1 h at RT (1x PBS, 5% normal goat serum, 0.3% Triton™ X-100). After blocking, IECs were incubated for 4 h with Alexa Fluor 488 conjugated anti ZO-1, claudin-1, or occludin (dilution 1:100 in PBS with 1% BSA and 0.3% Triton X-100) or overnight with unconjugated primary antibodies against E-cadherin. Next day, upon washing the slides (3x, PBS), those incubated with unconjugated primary antibodies were probed with either a secondary anti-rabbit or anti-mouse Alexa Fluor 488 or Alexa Fluor 546 conjugated antibody and ActinRed 555 ReadyProbes Reagent for actin staining (Molecular Probes, Thermo Fisher Scientific). All antibodies were purchased from Thermo Fisher Scientific, Claudin-1 cat no. 37–4900, Occludin cat no. 33–1500, E-cadherin cat. no. PA5-32178. Cells were washed with PBS containing a DAPI nuclear stain (Vector, cat no. H1200) and mounted using the VectaShield reagents (Vector, cat no. H1200). Images were taken using a Zeiss Axioplan II Imaging fluorescence microscope (Carl Zeiss Light Microscopy, ZEISS) and analyzed by ZEN 2.1 software.

Recombinant CPs (2.5 µg/ml) were each added to Caco-2 cells in the presence or absence of E64 (10 µM) and a control (Caco-2 cells in DMEM without the CPs) and incubated at 37°C for 24 h (10% CO_2_). Next day, cells were washed with PBS and fixed in 4% PFA prepared in PBS at room temperature for 15 min. Following fixation, cells were washed with PBS (3x), and blocked in 3% BSA in PBS at room temperature for 1 h. Cells were incubated over night with primary antibodies against occludin (1:100), claudin-4 (1:100), E-cadherin (1:100) and beta-catenin (1:50), all diluted in the blocking solution (3% BSA in PBS). Then, cells were washed with PBS (3x) and incubated with AlexaFluor 488 or Alexafluor-594-conjugated anti-rabbit or anti-mouse antibodies (1:200) (Thermo Fisher Scientific, cat no. A11008, A11029, A21207, A11005), at room temperature for 1 h. Cells were mounted and imaged as above.

### Western blot analysis of the effect of recombinant CPs on AJCs

Recombinant CPs (CP14019, CP16160 and CP16779) were added separately to Caco-2 cells at different concentrations (0–30 µg/ml) and incubated for 24 h at 37°C (10% CO_2_). Cells incubated in media alone served as a negative control. Next day, Caco-2 cells were washed twice with PBS, scraped off and the cell suspension was centrifuged (10 min at 500 *×* g, at 4°C) to pellet the cells for whole protein extraction. Pelleted cells were lyzed in NP-40 lysis buffer (Thermo Fisher Scientific, cat no. FNN0021) supplemented with Halt Protease Inhibitor Cocktail (Thermo Fisher, cat no. 78439) and stored at -80°C until use. Protein concentration was determined using the Bradford assay (Bio-Rad, cat no. 5000205) and equal amount of Caco-2 cell lysates (20 µg) were separated under reducing conditions on Any kDa Precast Tris-glycine gels (Bio-Rad, cat no. 4568123). Following SDS-PAGE, proteins were transferred to a nitrocellulose membrane (Pall Life Sciences, cat no. BSP0161) at 100 v for 1 h and blocked in Tris-buffered Saline containing 0.1% Tween-20 (TBST) and 3% BSA for 1 h at room temperature. Membranes were incubated overnight at 4°C with primary antibodies against Claudin-4 (1:5000 dilution), Occludin, E-cadherin, β-catenin (all at 1:2500 dilution), JAM-1 (1:10000) and GAPDH (dilution). All antibodies were purchased from Thermo Fisher Scientific, Claudin-1 cat no. 37–4900, Claudin-4 cat no. 32–9400, Occludin cat no. 33–1500, E-cadherin cat. no. PA5-32178, β-catenin cat no. 71–2700, JAM-1 cat no. 36–1700, GAPDH cat no. PA1-987 and MA5-15738. Next day membranes were washed three times in TBST and incubated with anti-rabbit or anti-mouse HRP-conjugated secondary antibodies (1:10000) and developed using the ECL kit (Bio-Rad, cat no. 170–5061). Membranes Signals were detected using a ChemiDoc MP System (Bio-Rad) and protein abundance was quantified using the ImageJ software and GAPDH as a loading control.

### *In vitro* degradation of rhEcadherin and rhOccludin

To test the direct proteolytic activity of CPs, different concentrations of mature recombinant proteins were each incubated with the recombinant human E-cadherin protein (rhE-cadherin; Advanced BioMatrix, cat no. 5085-0.1 MG) for up to 1 h at 37°C. Reaction mixtures contained 1 µg of recombinant E-cadherin and 200 nM of each CP, incubated for 0, 5, 10, 30, and 60 min. In addition, parallel reactions were prepared in which CPs were treated with 100 µM of E64 at 37°C for 30 min before incubation with rhE-cadherin. Samples were then separated under reducing conditions on any kDa precast Tris-glycine gels (Bio-Rad). To test the direct proteolytic activity of CPs on Occludin, 200 ng mature recombinant proteins were each incubated with 800 ng recombinant human Occludin protein (rhOccludin; Abcam, cat no. ab114189) for 30 min at 37°C. Parallel reactions were prepared in which CPs were treated with 100 µM of E64 at 37°C for 30 min before incubation with rhOccludin. Reactions were stopped by Laemmli sample buffer supplemented with beta-mercaptoethanol. Samples were analyzed by 4–12% bis-tris gels (Thermo Fisher Scientific, cat no. NP0329BOX) under reducing conditions. All gels were stained with QC Colloidal Coomassie Blue (Bio-Rad, cat no. 1610803) and visualized using a ChemiDoc MP System (Biorad).

### Proteolytic cleavage of chemokines

A range of proinflammatory cytokines and chemokines were incubated with *Giardia* ESPs to determine ESPs' ability to modulate immune response represented by cytokine degradation. The human recombinant cytokines Interlukin-8 (IL-8), chemokine ligand-20 (CCL20) and TNF-α were purchased from Sigma-Aldrich whereas chemokine ligand 1 and 3 (CXCL1 and CXCL3) as well as interleukin 1-alpha (IL-1-α) were purchased from R&D Signaling (R&D Systems). All cytokines were suspended in a sterile PBS at a concentration of 100 ng/μl. ESPs were collected from trophozoites in axenic culture in serum-free medium as described earlier and were quantified for total proteins using the Qubit protein assay reagent (Thermo Fisher Scientific). Next, 1μg of ESPs, from each isolate, was mixed with each one of the cytokines above and incubated at 37°C for 1 h. Cytokines or ESPs incubated alone were used as controls. For IL-8 and CCL20 only, we tested the effect of increasing ESPs concentration (1, 5 and 10 μg) or incubation time (1 h and 2 h) on cytokine degradation. At the end of all incubations, samples were mixed with Laemmli sample buffer, boiled for 5 min, cooled on ice and loaded onto Any kD™ Mini-PROTEAN® TGX Stain-Free™ Protein Gels (Bio-Rad) alongside Spectra™ Multicolor Broad Range Protein Ladder (Thermo Fisher Scientific). Gels were electrophoresed at 100 volts until the dye reached the bottom of the gel. Gels were fixed and stained using a QC Colloidal Commassie Stain (Bio-Rad) and visualised using a ChemiDoc Imaging System (BioRad).

Recombinant CPs were incubated with similar set of chemokines to test for chemokine degradation. The recombinant CPs (500 ng) were activated as described before (see the detection of cathepsin cysteine protease activity section) and incubated with 1 µg of recombinant human CXCL1, CXCL2, CXCL3, and CCL20 (Peprotech, CXCL1, cat no. 300–11, CXCL2, cat no. 300–39, CXCL3, cat no. 300–40, CCL20, cat no. 300–29A) for 60 min. For CCL2 and IL-8 (Peprotech, cat no. CCL2, cat no. 300–04, IL-8, cat no. 200–08 M), 1 µg of each was incubated with 500 ng of 14019 and 16779 for different durations ranging from 0 to 15 min. All reactions included chemokines with no CPs as a negative control. Parallel reactions with CPs treated with E-64 (100 µM) for 30 min (37°C) were setup as controls on CPs activity. At the end of all incubations, the reactions were stopped by the addition of 4 x Laemmli´s sample buffer (Bio-Rad, cat no. 1610747) and heating at 95°C for 5 min. Samples were electrophoresed on a 16.5% Tris-Tricine SDS-PAGE (Bio-Rad, cat no. 4563063) under reducing condition, and stained by QC Colloidal Coomassie Blue (Bio-Rad).

## Supplementary Material

1451284_supp.zip

## References

[1] Ankarklev J, Jerlström-Hultqvist J, Ringqvist E, et al. Behind the smile: Cell biology and disease mechanisms of *Giardia* species. Nat Rev microbiol. 2010;8:413–22. 20400969 10.1038/nrmicro2317

[2] Halliez MC, Buret AG Extra-intestinal and long term consequences of *Giardia* duodenalis infections. World J Gastroenterol. 2013;19:8974–85. 24379622 10.3748/wjg.v19.i47.8974 PMC3870550

[3] Certad G, Viscogliosi E, Chabe M, et al. Pathogenic mechanisms of *Cryptosporidium* and *Giardia*. Trends Parasitol. 2017;33:561–76. 28336217 10.1016/j.pt.2017.02.006

[4] Dawson SC An insider's guide to the microtubule cytoskeleton of *Giardia*. Cell Microbiol. 2010;12:588–98. 20184590 10.1111/j.1462-5822.2010.01458.x

[5] Einarsson E, Ma'ayeh S, Svard SG An up-date on *Giardia* and giardiasis. Cur Opin Microbiol. 2016;34:47–52. 10.1016/j.mib.2016.07.019 27501461

[6] Mmbaga BT, Houpt ER *Cryptosporidium* and *giardia* infections in children: A REview. Pediatr clin North Am. 2017;64:837–50. 28734513 10.1016/j.pcl.2017.03.014

[7] Schumann M, Siegmund B, Schulzke JD, et al. Celiac disease: Role of the epithelial barrier. Cell Mol Gastroenterol Hepatol. 2017;3:150–62. 28275682 10.1016/j.jcmgh.2016.12.006 PMC5331784

[8] Allain T, Amat CB, Motta JP, et al. Interactions of *Giardi**a* sp. with the intestinal barrier: Epithelium, mucus, and microbiota. Tissue Barriers. 2017;5:e1274354. 28452685 10.1080/21688370.2016.1274354 PMC5362998

[9] Yano T, Kanoh H, Tamura A, Tsukita S Apical cytoskeletons and junctional complexes as a combined system in epithelial cell sheets. Annals of the New York Academy of Sciences 2017. 10.1111/nyas.13432 28763830

[10] Hardin JA, Buret AG, Olson ME, et al. Mast cell hyperplasia and increased macromolecular uptake in an animal model of giardiasis. J Parasitol. 1997;83:908–12. 9379297

[11] Troeger H, Epple HJ, Schneider T, et al. Effect of chronic *Giardia lamblia* infection on epithelial transport and barrier function in human duodenum. Gut. 2007;56:328–35. 16935925 10.1136/gut.2006.100198 PMC1856804

[12] Chavez B, Gonzalez-Mariscal L, Cedillo-Rivera R, et al. *Giardia lamblia*: *in vitro* cytopathic effect of human isolates. Exp Parasitol. 1995;80:133–8. 7821402 10.1006/expr.1995.1015

[13] Teoh DA, Kamieniecki D, Pang G, et al. *Giardia lamblia* rearranges F-actin and alpha-actinin in human colonic and duodenal monolayers and reduces transepithelial electrical resistance. J Parasitol. 2000;86:800–6. 10958459 10.1645/0022-3395(2000)086[0800:GLRFAA]2.0.CO;2

[14] Buret AG, Mitchell K, Muench DG, et al. *Giardia lamblia* disrupts tight junctional ZO-1 and increases permeability in non-transformed human small intestinal epithelial monolayers: Effects of epidermal growth factor. Parasitol. 2002;125:11–9. 10.1017/s0031182002001853 12166516

[15] Humen MA, Perez PF, Lievin-Le Moal V Lipid raft-dependent adhesion of *Giardia intestinalis* trophozoites to a cultured human enterocyte-like Caco-2/TC7 cell monolayer leads to cytoskeleton-dependent functional injuries. Cell Microbiol. 2011;13:1683–702. 21790940 10.1111/j.1462-5822.2011.01647.x

[16] Maia-Brigagao C, Morgado-Diaz JA, De Souza W *Giardia* disrupts the arrangement of tight, adherens and desmosomal junction proteins of intestinal cells. Parasitol Int. 2012;61:280–7. 22146155 10.1016/j.parint.2011.11.002

[17] Jimenez JC, Uzcanga G, Zambrano A, et al. Identification and partial characterization of excretory/secretory products with proteolytic activity in *Giardia intestinalis*. J Parasitol. 2000;86:859–62. 10958474 10.1645/0022-3395(2000)086[0859:IAPCOE]2.0.CO;2

[18] Rodriguez-Fuentes GB, Cedillo-Rivera R, Fonseca-Linan R, et al. *Giardia duodenalis*: analysis of secreted proteases upon trophozoite-epithelial cell interaction in vitro. Mem do Inst Oswaldo Cruz. 2006;101:693–6. 10.1590/s0074-02762006000600020 17072486

[19] de Carvalho TB, David EB, Coradi ST, et al. Protease activity in extracellular products secreted in vitro by trophozoites of *Giardia duodenalis*. Parasitol Res. 2008;104:185–90. 18797927 10.1007/s00436-008-1185-z

[20] Cotton JA, Amat CB, Buret AG Disruptions of host immunity and inflammation by *Giardia Duodenalis*: Potential consequences for co-infections in the Gastro-Intestinal Tract. Pathogens. 2015;4:764–92. 26569316 10.3390/pathogens4040764 PMC4693164

[21] Jimenez JC, Fontaine J, Grzych JM, et al. Antibody and cytokine responses in BALB/c mice immunized with the excreted/secreted proteins of *Giardia intestinalis*: The role of cysteine proteases. Ann Trop Med Parasitol. 2009;103:693–703. 20030993 10.1179/000349809X12502035776351

[22] Chin AC, Teoh DA, Scott KG, et al. Strain-dependent induction of enterocyte apoptosis by *Giardia lamblia* disrupts epithelial barrier function in a caspase-3-dependent manner. Infect Immun. 2002;70:3673–80. 12065509 10.1128/IAI.70.7.3673-3680.2002 PMC128105

[23] Bhargava A, Cotton JA, Dixon BR, et al. *Giardia duodenalis* surface cysteine proteases induce cleavage of the intestinal epithelial cytoskeletal protein villin via myosin light chain kinase. Plos One. 2015;10:e0136102. 26334299 10.1371/journal.pone.0136102 PMC4559405

[24] Cotton JA, Bhargava A, Ferraz JG, et al. *Giardia duodenalis* cathepsin B proteases degrade intestinal epithelial interleukin-8 and attenuate interleukin-8-induced neutrophil chemotaxis. Infect Immun. 2014;82:2772–87. 24733096 10.1128/IAI.01771-14 PMC4097641

[25] Gerbaba TK, Gupta P, Rioux K, et al. *Giardia duodenalis*-induced alterations of commensal bacteria kill *Caenorhabditis elegans*: A new model to study microbial-microbial interactions in the gut. Am j physiol Gastrointest Liver Physiol. 2015;308:G550–61. 25573177 10.1152/ajpgi.00335.2014 PMC4360045

[26] Beatty JK, Akierman SV, Motta JP, et al. *Giardia duodenalis* induces pathogenic dysbiosis of human intestinal microbiota biofilms. Int j Parasitol. 2017;47:311–26. 28237889 10.1016/j.ijpara.2016.11.010

[27] Manko A, Motta JP, Cotton JA, et al. *Giardia* co-infection promotes the secretion of antimicrobial peptides beta-defensin 2 and trefoil factor 3 and attenuates attaching and effacing bacteria-induced intestinal disease. Plos One. 2017;12:e0178647. 28622393 10.1371/journal.pone.0178647 PMC5473565

[28] Morrison HG, McArthur AG, Gillin FD, et al. Genomic minimalism in the early diverging intestinal parasite *Giardia lamblia*. Sci. 2007;317:1921–6. 10.1126/science.1143837 17901334

[29] DuBois KN, Abodeely M, Sakanari J, et al. Identification of the major cysteine protease of *Giardia* and its role in encystation. J Biol Chem. 2008;283:18024–31. 18445589 10.1074/jbc.M802133200 PMC2440617

[30] Ringqvist E, Avesson L, Söderbom F, et al. Transcriptional changes in *Giardia* during host-parasite interactions. Int J Parasitol. 2011;41:277–85. 21074536 10.1016/j.ijpara.2010.09.011

[31] Ma'ayeh SY, Brook-Carter PT Representational difference analysis identifies specific genes in the interaction of *Giardia duodenalis* with the murine intestinal epithelial cell line, IEC-6. Int J Parasitol. 2012;42:501–9. 22561399 10.1016/j.ijpara.2012.04.004

[32] Ferella M, Davids BJ, Cipriano MJ, et al. Gene expression changes during *Giardia*-host cell interactions in serum-free medium. Mol Biochem Parasitol. 2014;197:21–3. 25286381 10.1016/j.molbiopara.2014.09.007

[33] Einarsson E, Troell K, Hoeppner MP, et al. Coordinated changes in gene expression throughout encystation of *Giardia intestinalis*. Plos Neglected Trop Dis. 2016;10:e0004571. 10.1371/journal.pntd.0004571 PMC4807828 27015092

[34] Emery SJ, van Sluyter S, Haynes PA Proteomic analysis in *Giardia duodenalis* yields insights into strain virulence and antigenic variation. Proteomics. 2014;14:2523–34. 25266764 10.1002/pmic.201400144

[35] Emery SJ, Mirzaei M, Vuong D, et al. Induction of virulence factors in *Giardia duodenalis* independent of host attachment. Sci Rep. 2016;6:20765. 26867958 10.1038/srep20765 PMC4751611

[36] Ward W, Alvarado L, Rawlings ND, et al. A primitive enzyme for a primitive cell: The protease required for excystation of *Giardia*. Cell. 1997;89:437–44. 9150143 10.1016/s0092-8674(00)80224-x

[37] Abodeely M, DuBois KN, Hehl A, et al. A contiguous compartment functions as endoplasmic reticulum and endosome/lysosome in *Giardia lamblia*. Eukaryot Cell. 2009;8:1665–76. 19749174 10.1128/EC.00123-09 PMC2772394

[38] Ma'ayeh S, Liu J, Peirasmaki D, et al. Characterization of the *Giardia intestinalis* secretome during interaction with human intestinal epithelial cells: the impact on host cells. Plos Neglected Tropical Diseases. 2017;11:e0006120. 29228011 10.1371/journal.pntd.0006120 PMC5739509

[39] DuBois KN, Abodeely M, Sajid M, et al. *Giardia lamblia* cysteine proteases. Parasitol Res. 2006;99:313–6. 16598471 10.1007/s00436-006-0149-4

[40] Halliez MC, Motta JP, Feener TD, et al. *Giardia duodenalis* induces paracellular bacterial translocation and causes postinfectious visceral hypersensitivity. Am j physiol Gastrointest and liver physiol. 2016;310:G574–85. 26744469 10.1152/ajpgi.00144.2015 PMC4836132

[41] Roxström-Lindquist K, Ringqvist E, Palm D, et al. *Giardia lamblia*-induced changes in gene expression in differentiated Caco-2 human intestinal epithelial cells. Infect Immun. 2005;73:8204–8. 16299316 10.1128/IAI.73.12.8204-8208.2005 PMC1307045

[42] Dubourg A, Xia D, Winpenny JP, et al. *Giardia* secretome highlights secreted tenascins as a key component of pathogenesis. GigaScience. 2018 10.1093/gigascience/giy003 PMC5887430 29385462

[43] Mach L, Mort JS, Glossl J Maturation of human procathepsin B. Proenzyme activation and proteolytic processing of the precursor to the mature proteinase, *in vitro*, are primarily unimolecular processes. J Biol Chem. 1994;269:13030–5. 8175723

[44] Pungercar JR, Caglic D, Sajid M, et al. Autocatalytic processing of procathepsin B is triggered by proenzyme activity. Febs J. 2009;276:660–8. 19143833 10.1111/j.1742-4658.2008.06815.x PMC4551429

[45] Kissoon-Singh V, Mortimer L, Chadee K *Entamoeba histolytica* cathepsin-like enzymes: Interactions with the host gut. Adv exp med biol. 2011;712:62–83. 21660659 10.1007/978-1-4419-8414-2_5

[46] Williams AG, Coombs GH Multiple protease activities in *Giardia intestinalis* trophozoites. Int J Parasitol. 1995;25:771–8. 7558562 10.1016/0020-7519(94)00201-x

[47] Coradi ST, Guimaraes S *Giardia duodenalis*: protein substrates degradation by trophozoite proteases. Parasitol Res. 2006;99:131–6. 16521040 10.1007/s00436-005-0124-5

[48] Pertuz Belloso S, Ostoa Saloma P, Benitez I, et al. *Entamoeba histolytica* cysteine protease 2 (EhCP2) modulates leucocyte migration by proteolytic cleavage of chemokines. Parasite Immunol. 2004;26:237–41. 15491473 10.1111/j.0141-9838.2004.00706.x

[49] Oberhuber G, Stolte M Histologic detection of trophozoites of *Giardia lamblia* in the terminal ileum. Scand J Gastroenterol. 1995;30:905–8. 8578191 10.3109/00365529509101599

[50] Oberhuber G, Kastner N, Stolte M Giardiasis: A histologic analysis of 567 cases. Scand J Gastroenterol. 1997;32:48–51. 9018766 10.3109/00365529709025062

[51] Oberhuber G, Mesteri I, Kopf W, et al. Demonstration of trophozoites of *G. lamblia* in ileal mucosal biopsy specimens may reveal giardiasis in patients with significantly inflamed parasite-free duodenal mucosa. Am J Surg Pathol. 2016;40:1280–5. 27186850 10.1097/PAS.0000000000000665

[52] Keister DB Axenic culture of *Giardia lamblia* in TYI-S-33 medium supplemented with bile. Trans R Soc Trop Med Hyg. 1983;77:487–8. 6636276 10.1016/0035-9203(83)90120-7

[53] Jerlström-Hultqvist J, Stadelmann B, Birkestedt S, et al. Plasmid vectors for proteomic analyses in *Giardia*: purification of virulence factors and analysis of the proteasome. Eukaryot Cell. 2012;11:864–73. 22611020 10.1128/EC.00092-12 PMC3416501

[54] Jerlström-Hultqvist J, Einarsson E, Svärd SG Stable transfection of the diplomonad parasite *Spironucleus salmonicida*. Eukaryot Cell. 2012;11:1353–61. 22983987 10.1128/EC.00179-12 PMC3486028

