# Supplementary material for: Secreted Giardia intestinalis cysteine proteases disrupt intestinal epithelial cell junctional complexes and degrade chemokines
Source: Virulence. 2018 May 4;9(1):879–94. doi: 10.1080/21505594.2018.1451284 (PMC5955458; doi:10.1080/21505594.2018.1451284)
Supplement: 1451284_supp.zip [file kvir-09-01-1451284-s001.zip › 1451284_supp/2017VIRULENCE0277R2-s02.docx]

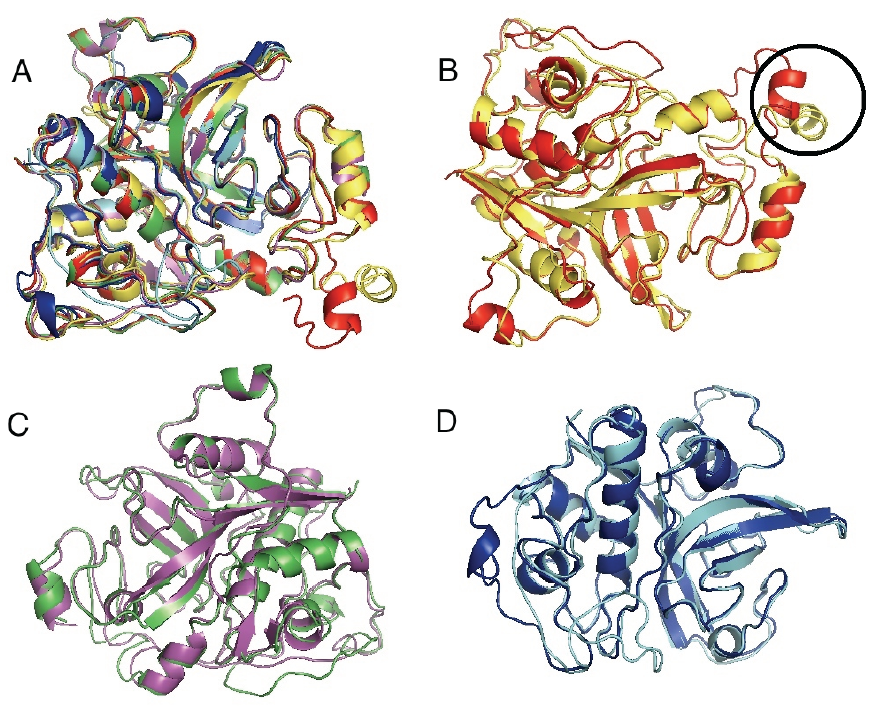


**Figure S1.** Superimposition of the CP14019 structure modeled by Phyre2 and I-TASSER. Phyre2 models in blue and I-TASSER models in red. **A.** Superimposition of all the models from both platforms. **B.** Superimposition of the models with the propeptides; RMS=0.689. Red-I-TASSER, Yellow-Phyre2. N-terminal helix in circle. **C.** Superimposition of the models of the proteases without signal peptide; RMS=0.580. Green-I-TASSER, Purple-Phyre2. **D.** Models of mature CP14019. RMS=0.438. Blue-I-TASSER, Green, Phyre2.
