# Supplementary material for: Secreted Giardia intestinalis cysteine proteases disrupt intestinal epithelial cell junctional complexes and degrade chemokines
Source: Virulence. 2018 May 4;9(1):879–94. doi: 10.1080/21505594.2018.1451284 (PMC5955458; doi:10.1080/21505594.2018.1451284)
Supplement: 1451284_supp.zip [file kvir-09-01-1451284-s001.zip › 1451284_supp/2017VIRULENCE0277R2-s03.docx]

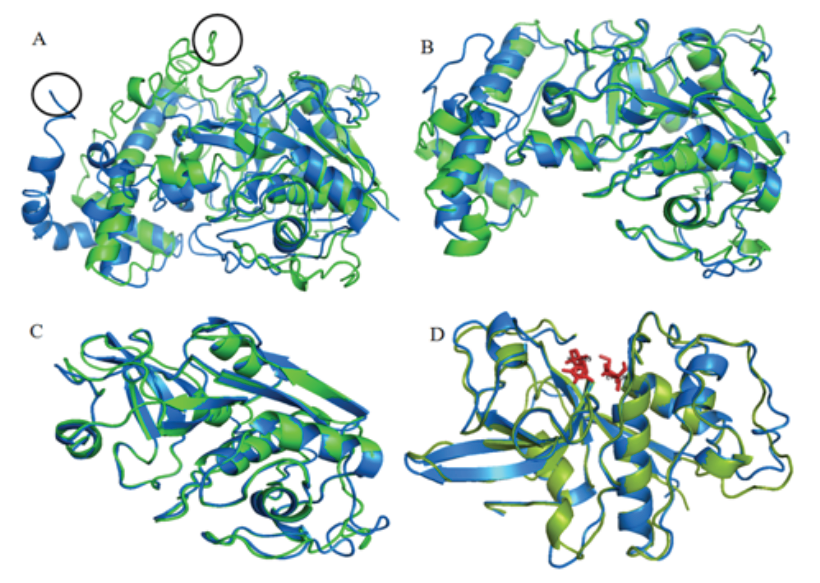


**Figure S2.** Superimposition of the CP16160 protein structure modeled by Phyre2 and I-TASSER Phyre2 models in blue and I-TASSER models in green. **A.** Superimposition of the models done with the entire CP16160 sequence, the N terminals are marked by a circle.. RMS=2.091. **B.** Superimposition of the models lacking the signal sequences; RMS=0.875. **C.** Superimposition of the models of the mature proteases; RMS=0.364. **D.** Models of mature CP16160 with catalytic residues labeled in red (Cys26 and His165).
