# Supplementary material for: Secreted Giardia intestinalis cysteine proteases disrupt intestinal epithelial cell junctional complexes and degrade chemokines
Source: Virulence. 2018 May 4;9(1):879–94. doi: 10.1080/21505594.2018.1451284 (PMC5955458; doi:10.1080/21505594.2018.1451284)
Supplement: 1451284_supp.zip [file kvir-09-01-1451284-s001.zip › 1451284_supp/2017VIRULENCE0277R2-s04.docx]

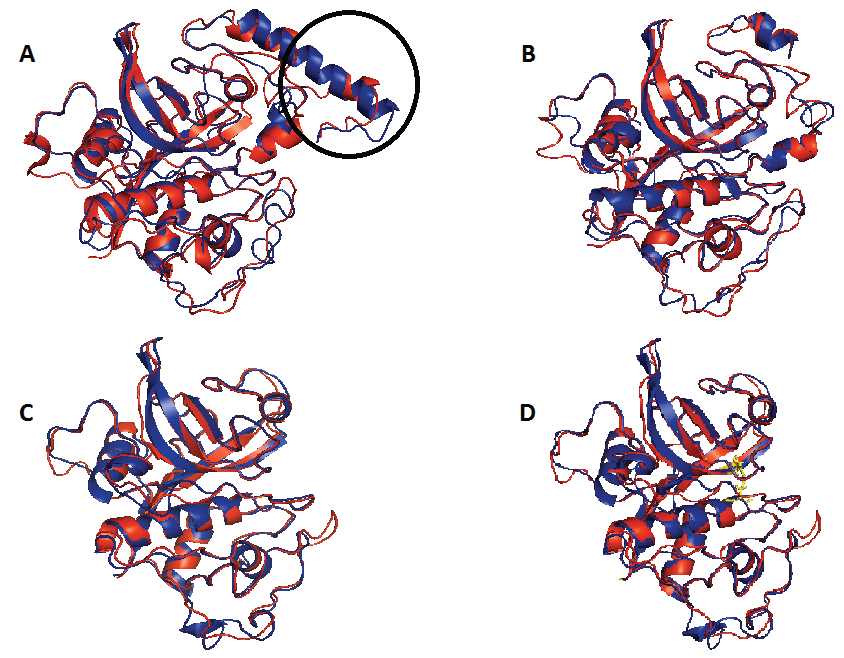


**Figure S3.** Superimposition of the CP16779 structure modeled by Phyre2 and I-TASSER. Phyre2 models in blue and I-TASSER models in red. **A.** Superimposition of the models done with the entire CP16779 sequence, including the pro-peptide. The N terminal helix marked by a circle. RMS=0.750. **B.** Superimposition of the models lacking the signal sequences; RMS=0.433. **C.** Superimposition of the models of the mature proteases; RMS=0.427. **D.** Models of mature CP16779 with predicted catalytic residues labeled in yellow.
