# Supplementary material for: Secreted Giardia intestinalis cysteine proteases disrupt intestinal epithelial cell junctional complexes and degrade chemokines
Source: Virulence. 2018 May 4;9(1):879–94. doi: 10.1080/21505594.2018.1451284 (PMC5955458; doi:10.1080/21505594.2018.1451284)
Supplement: 1451284_supp.zip [file kvir-09-01-1451284-s001.zip › 1451284_supp/2017VIRULENCE0277R2-s06.docx]

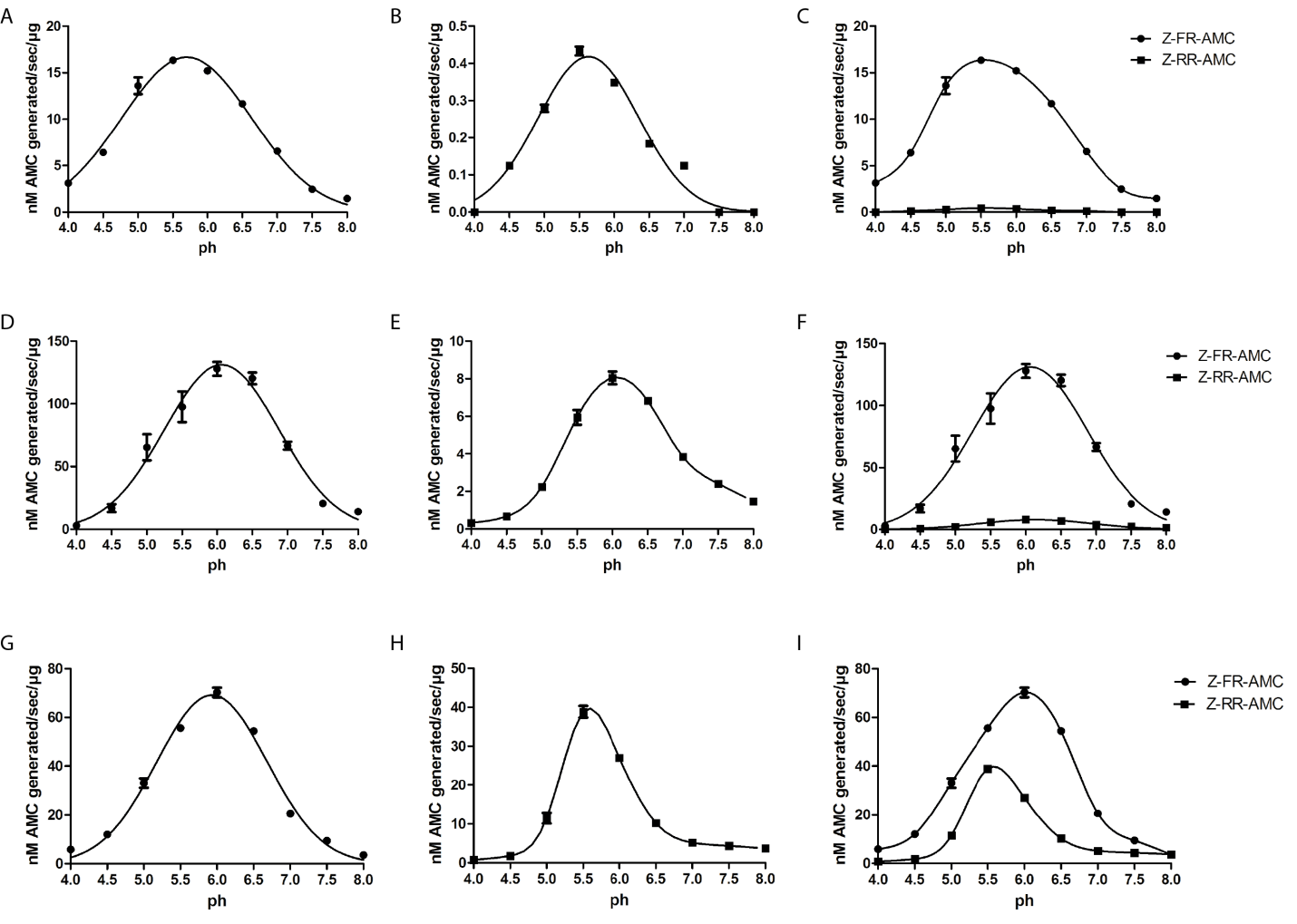


**Figure S5.** Optimal pH profiles of recombinant *G. intestinalis* secreted cysteine proteases. The same amount of enzyme was incubated in buffers with variant pH, fluorescence units were monitored over times at 37 ℃. Panels A, B, C showed that the optimal pH of mature enzyme 14019 against substrates Z-FR-AMC (A) and Z-RR-AMC (B) were found to be 5.5. Panels D, E, F showed that the optimal pH of mature enzyme 16779 against substrates Z-FR-AMC (D) and Z-RR-AMC (E) were found to be 6.0. Panels G, H, I showed that the optimal pH of mature enzyme 16160 against substrates Z-FR-AMC (G) and Z-RR-AMC (H) were found to be 6.0 and 5.5, respectively. All enzymes showed a preference for the Z-FR-AMC substrate.
