# Supplementary material for: Secreted Giardia intestinalis cysteine proteases disrupt intestinal epithelial cell junctional complexes and degrade chemokines
Source: Virulence. 2018 May 4;9(1):879–94. doi: 10.1080/21505594.2018.1451284 (PMC5955458; doi:10.1080/21505594.2018.1451284)
Supplement: 1451284_supp.zip [file kvir-09-01-1451284-s001.zip › 1451284_supp/2017VIRULENCE0277R2-s07.docx]

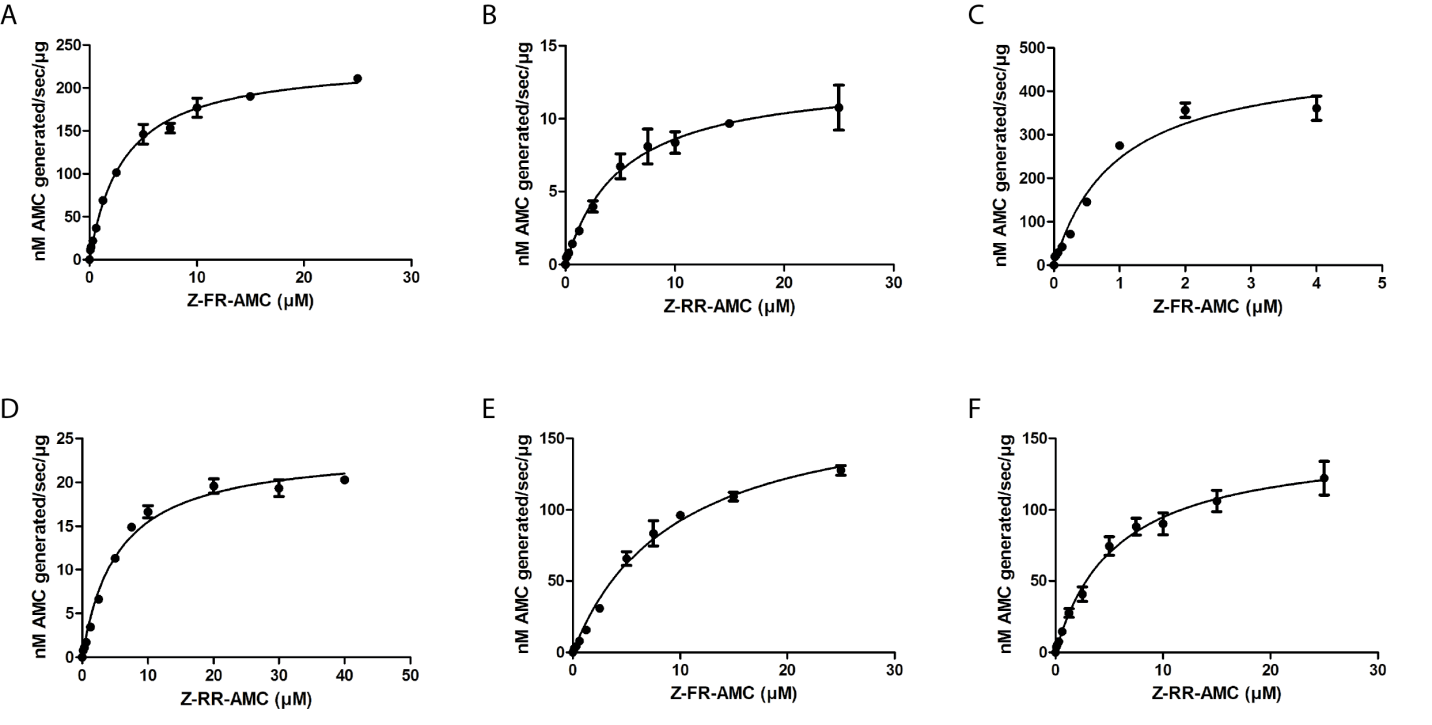


**Figure S6.** Kinetics measurements of recombinant *G. intestinalis* secreted cysteine proteases. The same amount of active enzyme was incubated with variant concentrations of substrates under the optimum pH, fluorescence units were monitored over times at 37 ℃. Panels A and B show the Michaelis-Menten curve of 14019 against substrates Z-FR-AMC (A) and Z-RR-AMC (B). Panels C and D show the Michaelis-Menten curve of 16779 against substrates Z-FR-AMC (C) and Z-RR-AMC (D). Panels E and F show the Michaelis-Menten curve of 16160 against substrates Z-FR-AMC (E) and Z-RR-AMC (F).
