# Supplementary material for: Secreted Giardia intestinalis cysteine proteases disrupt intestinal epithelial cell junctional complexes and degrade chemokines
Source: Virulence. 2018 May 4;9(1):879–94. doi: 10.1080/21505594.2018.1451284 (PMC5955458; doi:10.1080/21505594.2018.1451284)
Supplement: 1451284_supp.zip [file kvir-09-01-1451284-s001.zip › 1451284_supp/2017VIRULENCE0277R2-s08.docx]

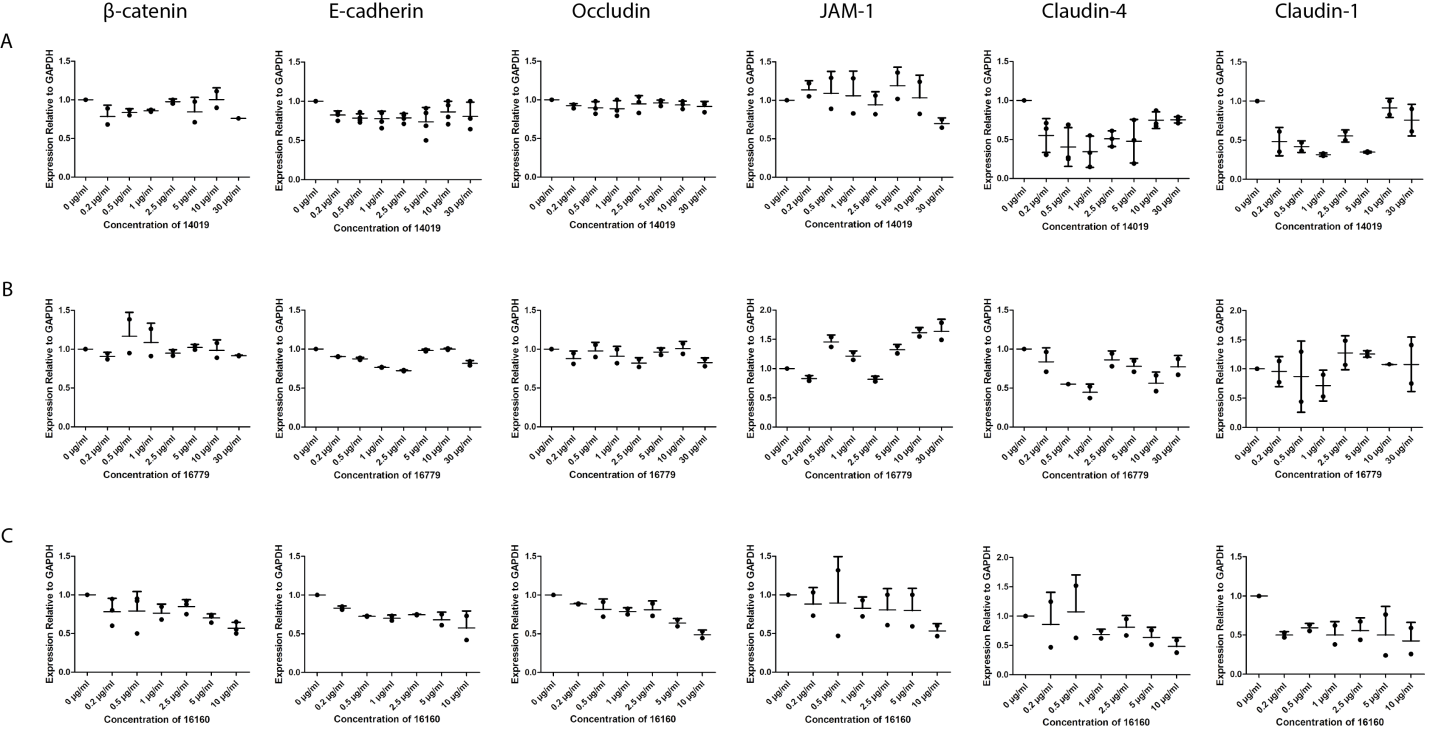


**Figure S7.** Quantification of bands in the Western blot shown in Figure 5. The intensity of Western blot bands of apical junctional proteins treated by 14019 (A), 16779 (B) and 16160 (C) were quantified using the Image J software relative to GAPDH. Standard deviations were calculated by Graphpad Prism 5.0. Biological triplicates for 14019 and biological duplicates for 16779 or 16160 were used to do the calculation.
