# Supplementary material for: Secreted Giardia intestinalis cysteine proteases disrupt intestinal epithelial cell junctional complexes and degrade chemokines
Source: Virulence. 2018 May 4;9(1):879–94. doi: 10.1080/21505594.2018.1451284 (PMC5955458; doi:10.1080/21505594.2018.1451284)
Supplement: 1451284_supp.zip [file kvir-09-01-1451284-s001.zip › 1451284_supp/2017VIRULENCE0277R2-s09.docx]

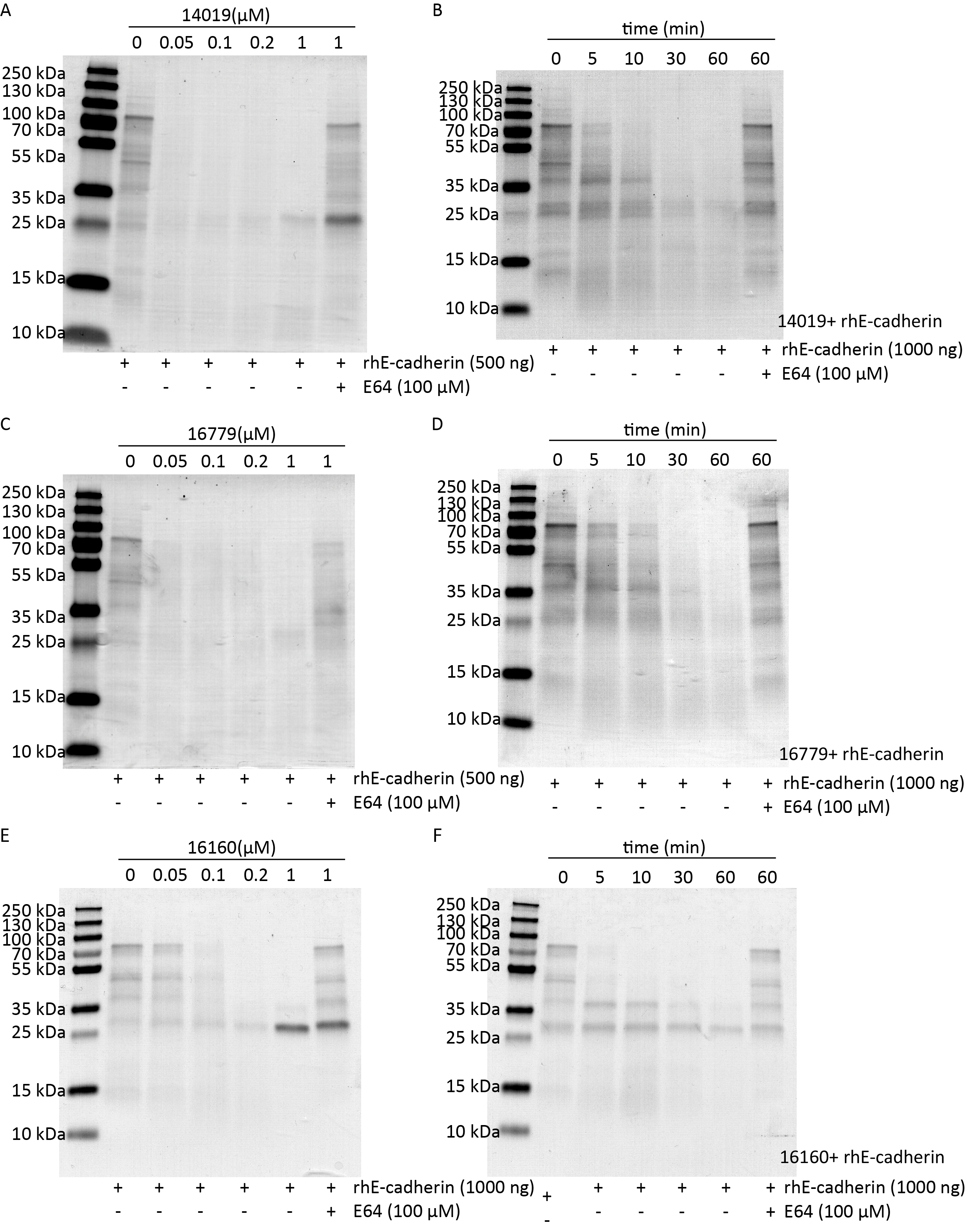


**Figure S8.** Cleavage of recombinant human E-cadherin protein (rhE-cadherin) by cysteine proteases. Different concentrations of recombinant cysteine proteases (A, 14019; C, 16779; E, 16160) were incubated with 500 ng or 1000 ng of rhE-cadherin for 1 h at 37 ℃. Recombinant cysteine proteases (0.2 µM) were incubated with 1000 ng of rhE-cadherin for different time points (0 min and 60 min) at 37 ℃ (B, 14019; D, 16779; F, 16160). Selected samples were routinely pretreated by 100 µM of E-64 for 30 min at 37 ℃ before incubate with rhE-cadherin. Sample proteins were separated by SDS-PAGE under reducing condition.
