# Supplementary material for: Secreted Giardia intestinalis cysteine proteases disrupt intestinal epithelial cell junctional complexes and degrade chemokines
Source: Virulence. 2018 May 4;9(1):879–94. doi: 10.1080/21505594.2018.1451284 (PMC5955458; doi:10.1080/21505594.2018.1451284)
Supplement: 1451284_supp.zip [file kvir-09-01-1451284-s001.zip › 1451284_supp/2017VIRULENCE0277R2-s10.docx]

**
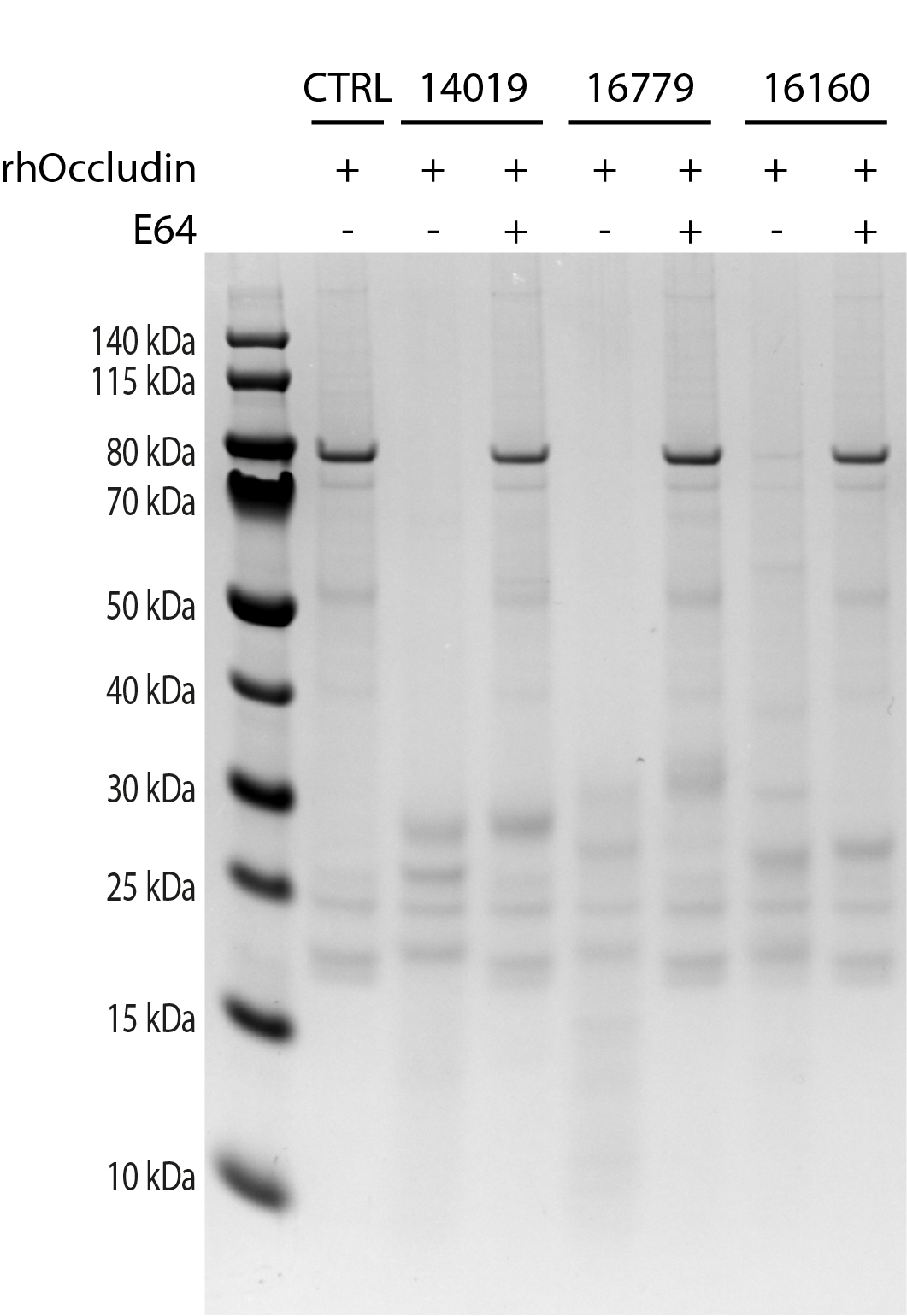
**

**Figure S9.** *G. intestinalis* CP digest recombinant human Occludin.

Cleavage of recombinant human Occludin by recombinant cysteine proteases was performed as described in Methods. 100 µM E64 was routinely added to the proteases for 30 min at 37 °C. Samples were analyzed by 4-12% Bis-Tris gel under reducing condition.
