# Supplementary material for: Secreted Giardia intestinalis cysteine proteases disrupt intestinal epithelial cell junctional complexes and degrade chemokines
Source: Virulence. 2018 May 4;9(1):879–94. doi: 10.1080/21505594.2018.1451284 (PMC5955458; doi:10.1080/21505594.2018.1451284)
Supplement: 1451284_supp.zip [file kvir-09-01-1451284-s001.zip › 1451284_supp/2017VIRULENCE0277R2-s13.docx]

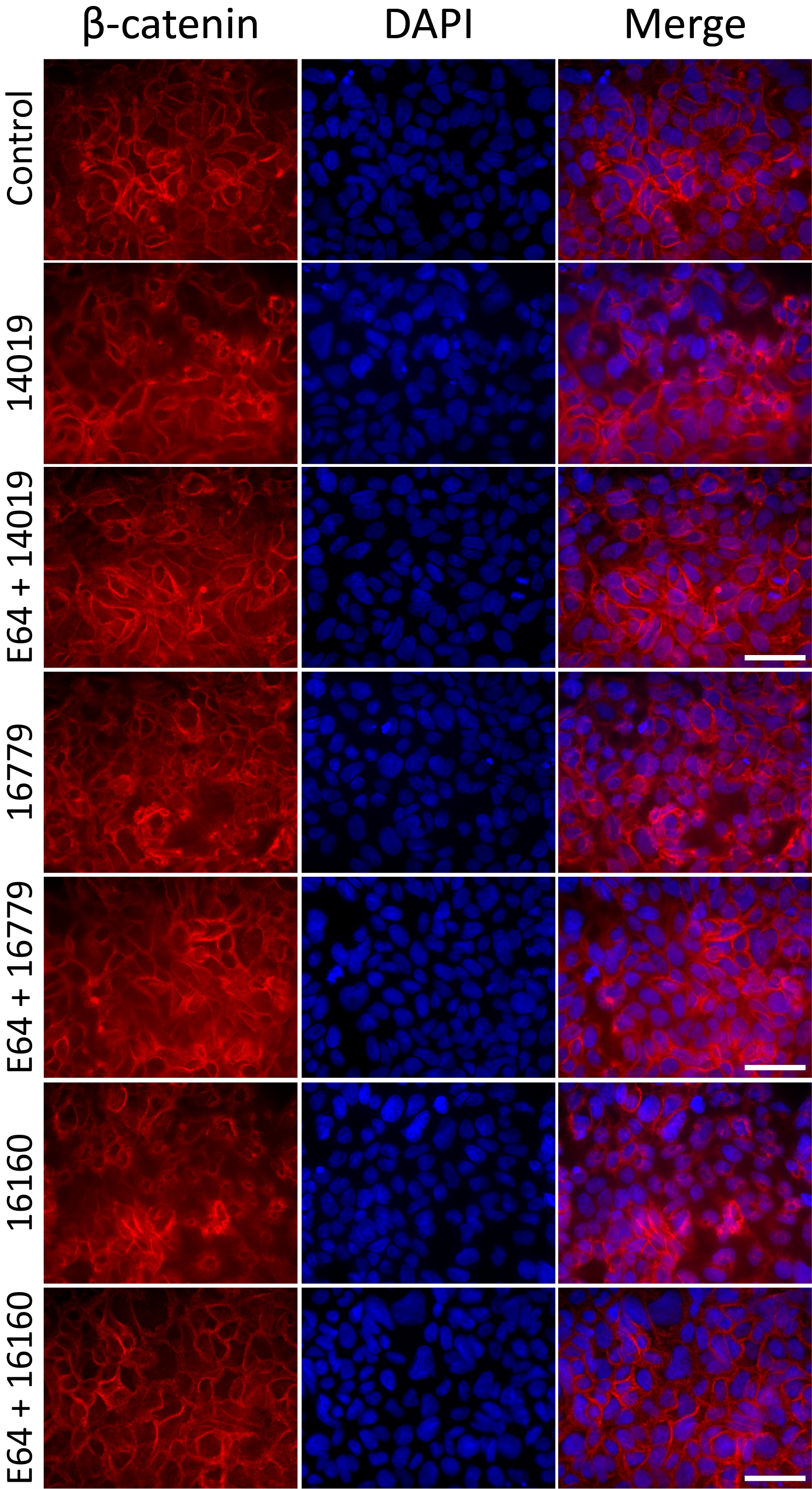


**Figure S12.** The expression and localization of β-catenin in Caco-2 cells visualized by immunofluorescence microscope. Caco-2 monolayers were incubated with 2.5 µg/ml of 14019, 16779 and 16160 in the absence or presence of E64 for 24 h, followed by fixation of PFA. β-catenin was detected with a rabbit anti- β-catenin antibody and then incubated with an Alexa Fluor 594 conjugated rabbit antibody. *Bar*, 50 µm. Images were taken by a Zeiss Axioplan II Imaging fluorescence microscope and analyzed by ZEN 2.1 software.
