# Supplementary figures and images for: Secreted Giardia intestinalis cysteine proteases disrupt intestinal epithelial cell junctional complexes and degrade chemokines
Source: Virulence. 2018 May 4;9(1):879–94. doi: 10.1080/21505594.2018.1451284 (PMC5955458; doi:10.1080/21505594.2018.1451284)

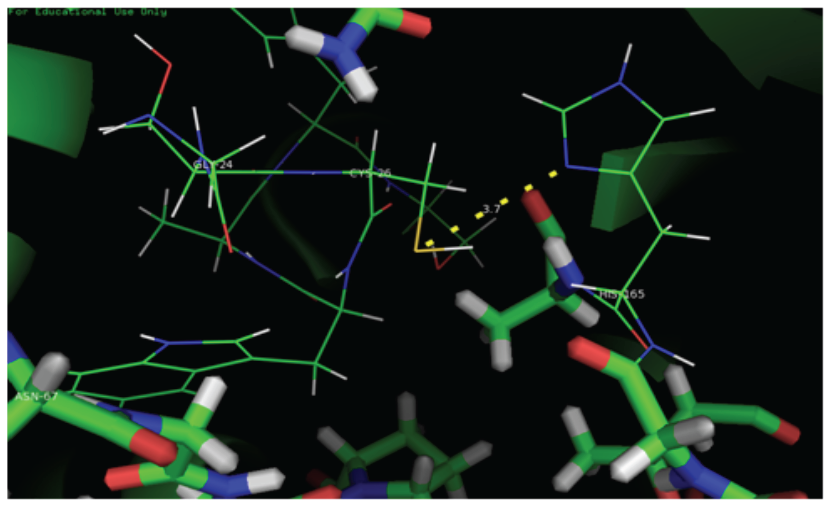


**Figure S4.** Predicted active site of the mature CP16160 protease. The distance between Cys26 and His 165 is 3.7 Å.

Supplement: 1451284_supp.zip [file kvir-09-01-1451284-s001.zip › 1451284_supp/2017VIRULENCE0277R2-s05.docx]

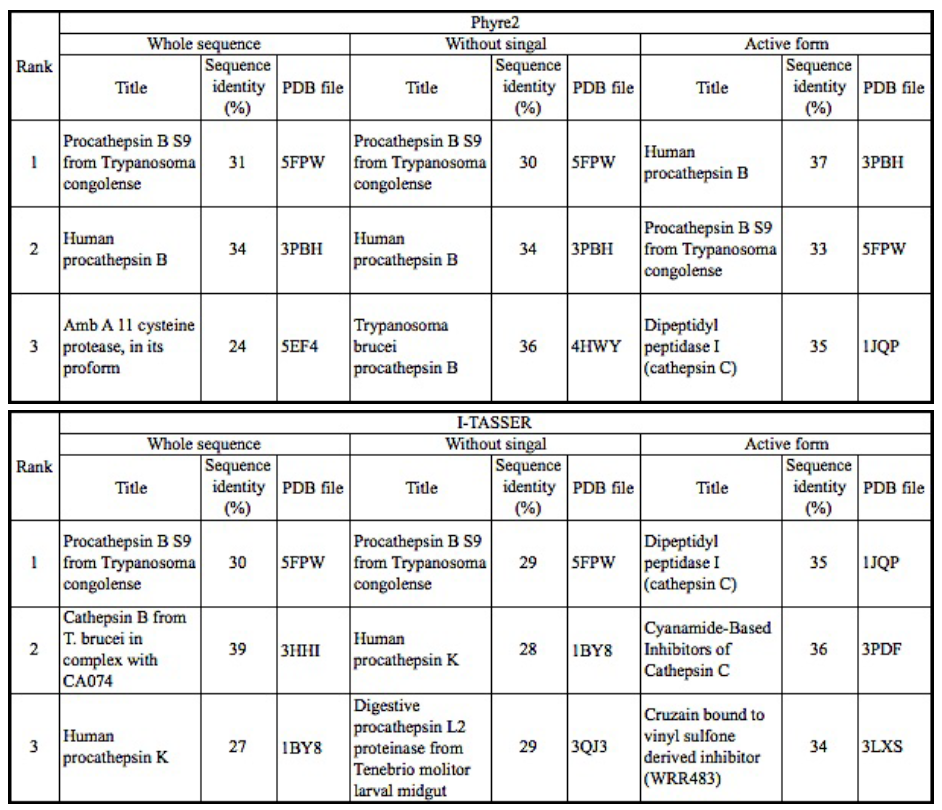


**Table S3.** PDB files used for homology modeling of CP14019 in Phyre2 and I-TASSER.

Supplement: 1451284_supp.zip [file kvir-09-01-1451284-s001.zip › 1451284_supp/2017VIRULENCE0277R2-s17.docx]

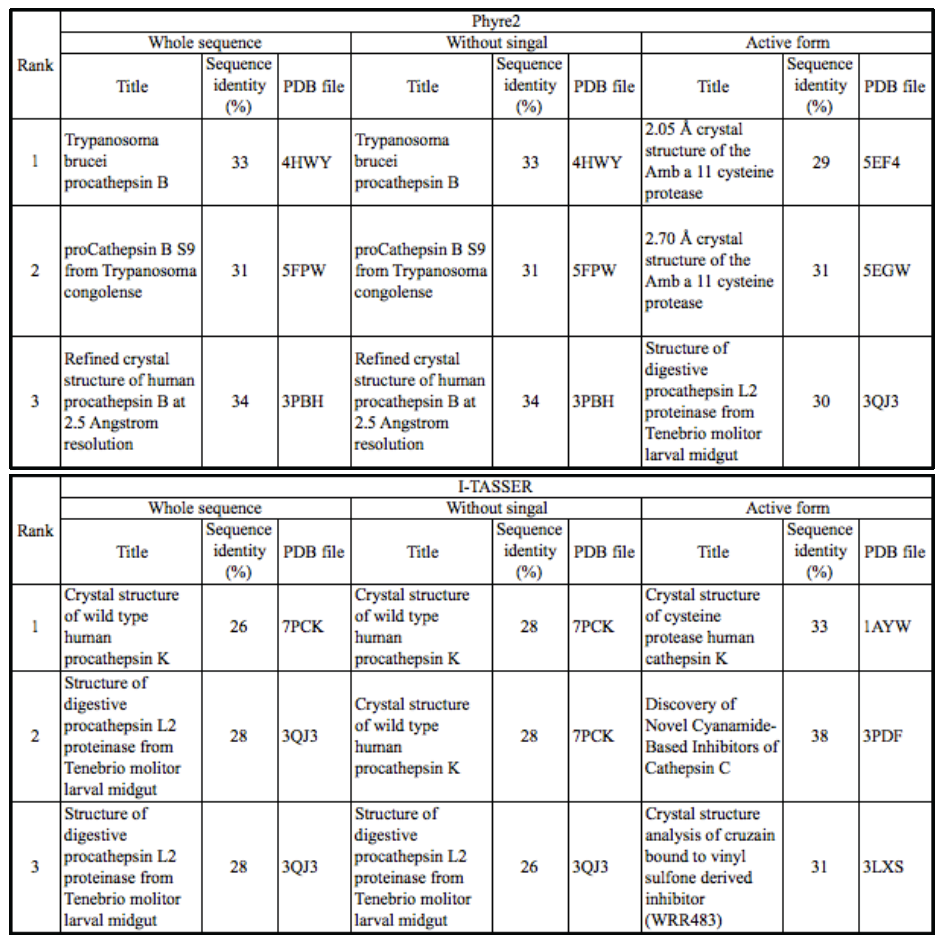


**Table S4.** PDB files used for homology modeling of CP16160 in Phyre2 and I-TASSER.

Supplement: 1451284_supp.zip [file kvir-09-01-1451284-s001.zip › 1451284_supp/2017VIRULENCE0277R2-s18.docx]

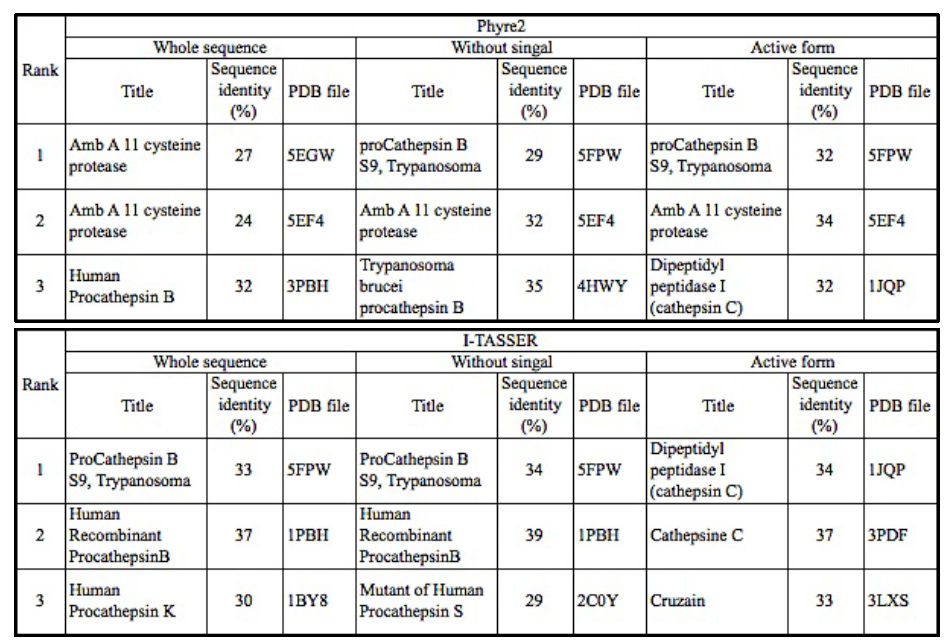


**Table S5.** PDB files used for homology modeling of CP16779 in Phyre2 and I-TASSER.

Supplement: 1451284_supp.zip [file kvir-09-01-1451284-s001.zip › 1451284_supp/2017VIRULENCE0277R2-s19.docx]
